# A Complex of BBS1 and NPHP7 Is Required for Cilia Motility in Zebrafish

**DOI:** 10.1371/journal.pone.0072549

**Published:** 2013-09-12

**Authors:** Yun Hee Kim, Daniel Epting, Krasimir Slanchev, Christina Engel, Gerd Walz, Albrecht Kramer-Zucker

**Affiliations:** 1 Renal Division, University Hospital Freiburg, Freiburg, Germany; 2 Spemann Graduate School of Biology and Medicine (SGBM), Albert-Ludwigs-University of Freiburg, Freiburg, Germany; 3 Faculty of Biology (or Faculty of Chemistry, Pharmacy, and Earth Sciences), Albert-Ludwigs-University of Freiburg, Freiburg, Germany; 4 Neurobiology, Max-Planck-Institute, Martinsried, Germany; Leibniz Institute for Age Research - Fritz Lipmann Institute (FLI), Germany

## Abstract

Bardet-Biedl syndrome (BBS) and nephronophthisis (NPH) are hereditary autosomal recessive disorders, encoded by two families of diverse genes. BBS and NPH display several overlapping phenotypes including cystic kidney disease, retinitis pigmentosa, liver fibrosis, *situs inversus* and cerebellar defects. Since most of the BBS and NPH proteins localize to cilia and/or their appendages, BBS and NPH are considered ciliopathies. In this study, we characterized the function of the transcription factor Nphp7 in zebrafish, and addressed the molecular connection between BBS and NPH. The knockdown of zebrafish *bbs1* and *nphp7.2* caused similar phenotypic changes including convergent extension defects, curvature of the body axis, hydrocephalus, abnormal heart looping and cystic pronephros, all consistent with an altered ciliary function. Immunoprecipitation assays revealed a physical interaction between BBS1 and NPHP7, and the simultaneous knockdown of z*bbs1* and z*nphp7.2* enhanced the cystic pronephros phenotype synergistically, suggesting a genetic interaction between z*bbs1* and z*nphp7.2 in vivo*. Deletion of zBbs1 or zNphp7.2 did not compromise cilia formation, but disrupted cilia motility. Although NPHP7 has been shown to act as transcriptional repressor, our studies suggest a crosstalk between BBS1 and NPHP7 in regulating normal function of the cilium.

## Introduction

Autosomal recessive cystic kidneys are typically part of complex syndromes involving multiple organs. Since most gene products mutated in these syndromes localize to the cilium, an abnormal function of the cilium has been implicated in their pathogenesis. Cilia are specialized microtubule-based organelles attached to most vertebrate cells. The primary cilium is thought to function as a mechano- and/or chemosensor, which detects signals from extracellular environment and transmits them to the inside of the cell. Mutations of numerous ciliary proteins lead to structurally or functionally abnormal cilia [1]. The resulting defects are closely related to developmental and degenerative disorders, collectively termed ‘ciliopathies’ [2]. In kidney epithelial cells, the primary cilium is involved in important signalling cascades such as Hedgehog, Wnt, planar cell polarity, and calcium-dependant signalling pathways [3]. Cilia-related cystic kidney diseases include autosomal dominant as well as recessive polycystic kidney disease, including the Bardet–Biedl syndrome (BBS) and nephronophthisis (NPH) [4].

Bardet-Biedl syndrome (BBS) is a genetically heterogeneous autosomal recessive disorder (*BBS 1-17*) [5], symptoms of which include obesity, retinal degeneration, anosmia, post-axial polydactyly, cognitive impairment, hypogenitalism, renal and cardiovascular anomalies, diabetes mellitus and hypertension [6], [7]. The BBS proteins have been reported to form a stable multiprotein complex called BBSome (BBS1, 2, 4, 5, 7, 8 and 9), and to play a role in ciliogenesis and cilia maintenance [8], [9], [10]. NPH is an autosomal recessive cystic kidney disease associated with retinal degeneration, cerebellar hypoplasia, liver fibrosis, *situs inversus*, and mental retardation [11], [12], [13]. Mutations in 15 genes have been linked to NPHP so far (OMIM 614845). The proteins encoded by *NPH* genes are called nephrocystins, and are highly conserved among species. They are involved in various cellular signalling events, employing the non-canonical Wnt, sonic hedgehog and planar cell polarity signalling pathways [11], [14]. Recent studies indicate that most of BBS and NPHP proteins localize to the cilia/basal body complex [15], [16], [17], [18], providing a structural basis for the overlap of the symptoms exhibited by BBS and NPH patients, including renal cysts and retinitis pigmentosa, liver fibrosis, *situs inversus* and mental problems, and supporting the notion that BBS and NPHP protein family share functional pathways [19], [20].

During the last decade zebrafish has emerged as a powerful animal system for studying ciliopathies [21], [22]. In the present study, we analyzed the biochemical interaction between BBS1 and NPHP7, and investigated this interaction in zebrafish. BBS1 is one of the seven members of the BBSome, which regulates vesicular trafficking of proteins to the ciliary membrane [10], [23]. Mutations of *BBS1* in humans have been reported as one of the most frequent causes of BBS, implying a significant function of BBS1 [6], [24], [25], [26]. BBS1 is highly expressed in the kidney [24]. NPHP7/GLIS2 (Gli-similar 2) is a member of the Gli-related Krüppel-like zinc-finger (ZF) transcription factor subfamily [27], [28]. Glis2 is essential for maintaining renal functions by regulating genes which are involved in epithelial-to-mesenchymal transition, fibrosis and apoptosis [29], [30]. In adult mouse kidneys, Glis2 was detected in epithelial cells of the renal tubule and Bowman's capsule [29]. *Glis2^lacZ/lacZ^* mutant mice developed renal atrophy, fibrosis and glomerular cysts, thus resembling some of the key features of NPH [29], [30]. Both BBS1 and GLIS2 are present in cilia [23], [29], [31].

Morpholino oligonucleotide (MO)-mediated knockdown of z*bbs1* and z*nphp7.2* in zebrafish embryos caused phenotypic changes characteristic for “ciliopathies”. Detailed analysis of cilia revealed that despite normal morphology, the cilia in the pronephric tubule and the nasal pit of zBbs1- and zNphp7.2-depleted embryos showed aberrant motility, suggesting that the observed phenotypes are due to the disruption of the normal ciliary beating pattern.

## Results

### BBS1 and NPHP7 interact with each other

**Figure 1:**
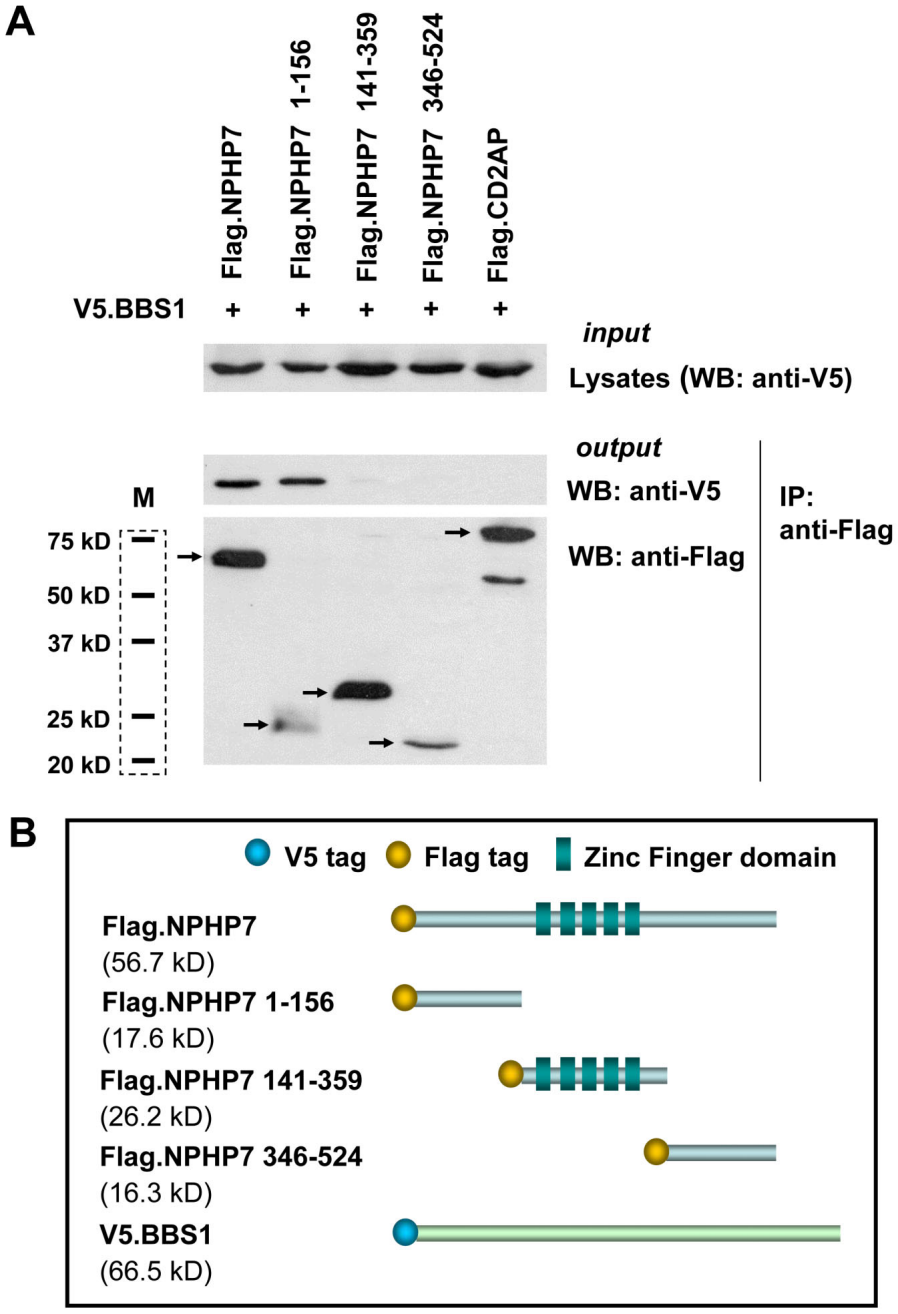
BBS1 interacts with N-terminus (amino acids 1–156) of NPHP7. (A) Full-length or FLAG-tagged truncation of NPHP7 (NP_115964.2) was co-expressed with V5-tagged BBS1 (V5.BBS1). Flag-tagged CD2AP (Flag.CD2AP) was used as a negative control. After precipitation using anti-Flag M2 agarose beads, the precipitates were analyzed for the presence of V5-tagged BBS1 with anti-V5 antibodies by western blotting. V5.BBS1 was precipitated by Flag.NPHP7 1–156. Arrows indicate precipitated Flag-tagged proteins. In the lane of CD2AP, there was an additional non-specific band at the position of 55 kD. The short black lines in the dashed box indicate protein size markers (M). (B) Schematic description of V5-tagged full-length BBS1, Flag-tagged full-length (Flag.NPHP7) and 3 different fragments (Flag.NPHP7 1–156, Flag.NPHP7 141–359, Flag.NPHP7 346–524) of NPHP7. Flag.NPHP7 141–359 contains 5 zinc finger (ZF) domains.

To investigate the interconnection between BBS and NPH protein families, we tested the interaction between NPHP7 and the BBS family members BBS1-12, using tagged versions of human proteins overexpressed in human embryonic kidney (HEK) 293T cells. We took interest in NPHP7 for the reason of being a transcription factor not having been very well characterized so far. Precipitation of BBS1 immobilized NPHP7 most extensively, suggesting that this interaction may be relevant *in vivo*. Since BBS1 has been characterized as a component of the BBSome, involved in ciliary transport processes, we decided to clarify the role of the BBS1/NPHP7 complex. Fig. 1A shows the interaction between human BBS1 and NPHP7. While BBS1 lacks apparent conserved functional domains, NPHP7 is a transcription factor with 5 zinc finger (ZF) domains responsible for DNA binding and transcriptional regulation [32]. To identify the domain of NPHP7 responsible for the interaction with BBS1, we generated three truncation constructs, Flag-NPHP7 1–156, Flag-NPHP7 141–359 and Flag-NPHP7 346–525 (Fig. 1B). We found that the N-terminus (Flag-NPHP7 1–156) of NPHP7 interacts with V5-BBS1 (Fig. 1A). Unfortunately, the lack of suitable antibodies for NPHP7 prevented us from confirming this interaction *in vitro*. Therefore, we turned to zebrafish, a genetically tractable animal model, to search for a potential genetic interaction between these two gene products.

### z*bbs1* and z*nphp7* are expressed in various tissues in the zebrafish

**Figure 2:**
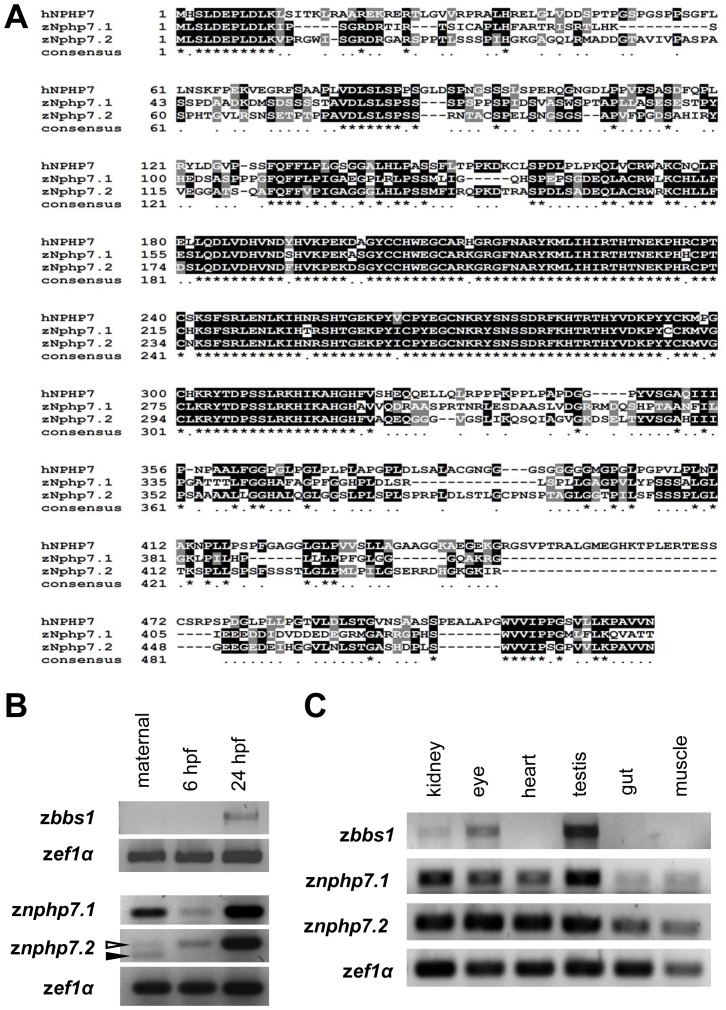
Expression of z*bbs1* and z*nphp7*. (A) Identification of 2 NPHP7 homologues in zebrafish: Zebrafish Nphp7.1 (zNphp7.1) and zebrafish Nphp7.2 (zNphp7.2) consist of 446 and 489 amino acids respectively. Amino acid sequence alignment showed that zNphp7.1 shares 43.9% identity and 50.8% similarity with the human NPHP7/GLIS2 (hNPHP7); zNphp7.2 was 51.4% identical and 60.2% similar to the human homologue. The ZF domains of zNphp7.1 and zNphp7.2 were 89.3% and 91.3% identical with those of the human homologue, respectively. (*, completely conserved; **.**, identical in 2 sequences or belonging to same type of amino acid group in 2 or 3 sequences) (B) Semi-quantitative RT-PCR reveals maternal transcript expression for z*nphp7.1* and z*nphp7.2* whereas z*bbs1* is not expressed maternally nor at 6 hpf. 2 maternal splice products were identified for z*nphp7.2* (open arrowhead: Transcript 1; filled arrowhead: Transcript 2). The transcript 2 of z*nphp7.2* is expressed only maternally. Sequencing of the lower splice product revealed an excision of 18 bp corresponding to amino acid (aa 101–118) (Fig. S1). (C) Semi-quantitative RT-PCR with organ specific cDNA from adult zebrafish indicated that z*bbs1* is expressed in kidney, eye and testis. z*nphp7.1* and z*nphp7.2* are expressed in other organs including kidney, eye, heart, testis, gut and muscle.

The zebrafish homologue of human BBS1 has been previously described [33]. Homology blast searches with the human protein as a query identified 2 potential zebrafish Nphp7 homologues. The genes encoding these proteins are located on chromosome 22 and 3, which we named *znphp7.1* (Genbank KF054060, Zv9: ENSDARG00000078388) and *znphp7.2* (Genbank KF054061, Zv9: ENSDARG00000073861), respectively; subsequent genome annotations (genome assembly ZV9) confirmed our findings. Protein alignments showed that zNphp7.1 sequence had 43.9% identity and 50.8% similarity to the human NPHP7/GLIS2; zNphp7.2 was 51.4% identical and 60.2% similar to the human gene product (Fig. 2A). As for the Gli family, the domain with the highest identity was the ZF domain: zNphp7.1 and zNphp7.2 were 89.3% and 91.3% identical with the human ZF domain of human GLIS2, respectively. Previous studies showed ubiquitous expression of *BBS1* in human organs including fetal tissue, testis, retina and adipose tissue with the highest expression in the kidney [24]. *Glis2* mRNA was also most abundantly expressed in mouse kidney with low levels in heart, lung, placenta, prostate, colon, and brain [27], [28]. In zebrafish whole mount in situ hybridization with various antisense RNA probes of z*bbs1* and z*nphp7.1* and z*nphp7.2* did not show consistently reproducible results. Semi-quantitative RT-PCR revealed that z*nphp7.1* and z*nphp7.2* are expressed maternally, at 6 hpf and 24 hpf whereas z*bbs1* is expressed at 24 hpf. A second maternal splice variant of z*nphp7.2* (Transcript 2) was observed and confirmed by sequencing (Fig. 2B and Fig. S1). The sequence alignment showed that 1 amino acid is substituted (S99R) and 18 amino acids (aa 101–118) are not coded for in transcript 2 of z*nphp7.2* (Fig. S1). In addition, semi-quantitative RT-PCR from various organs of adult zebrafish showed that z*bbs1*, z*nphp7.1* and z*nphp7.2* are expressed in kidney, eye and testis. However, z*nphp7.1* and z*nphp7.2* are additionally expressed in the heart, gut and muscle (Fig. 2C).

### Depletion of zBbs1 and zNphp7 in zebrafish embryos causes ciliopathic phenotypes including pronephric cyst formation

**Figure 3:**
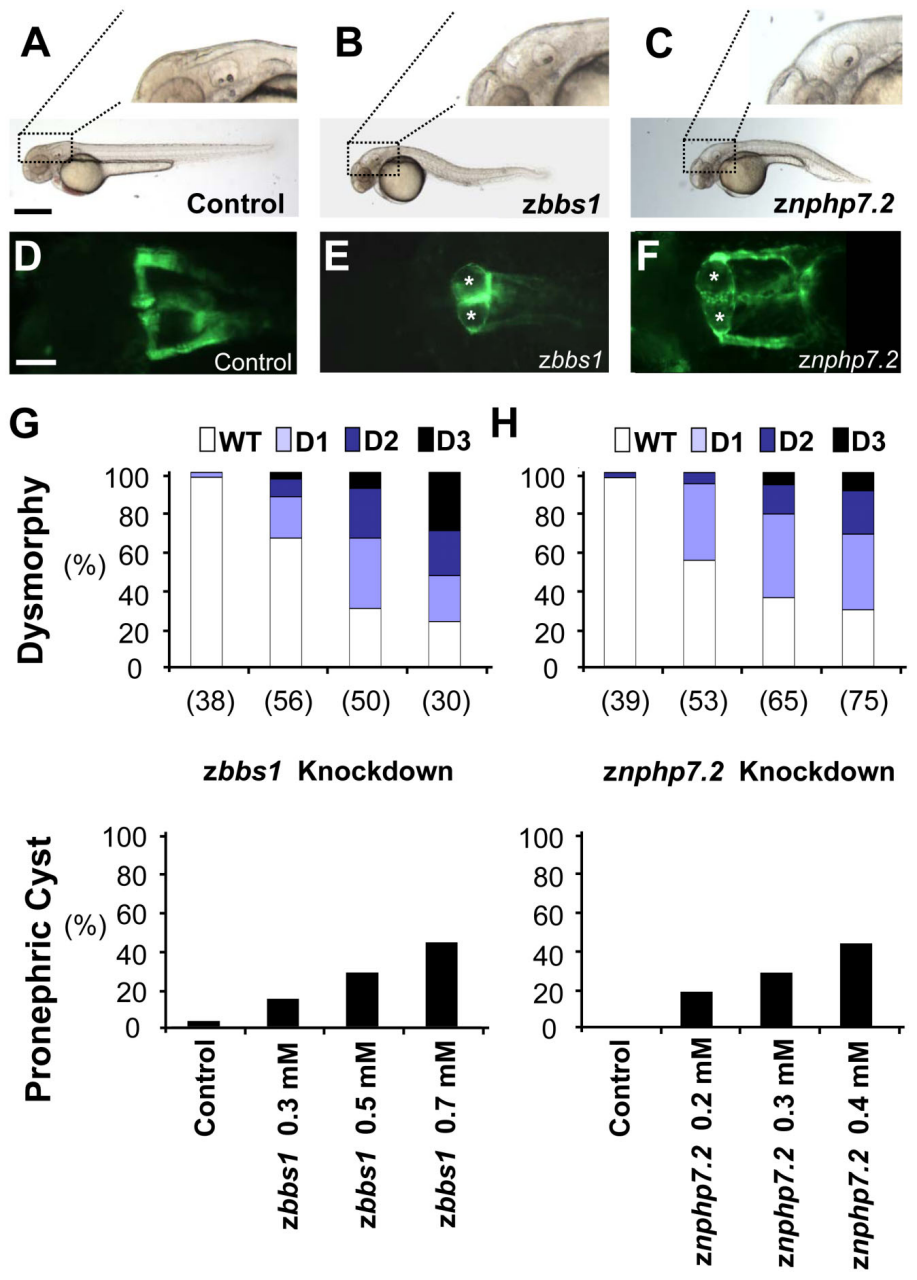
Depletion of zBbs1 and zNphp7.2 causes dose-dependent hydrocephalus and pronephric cysts. Zebrafish embryos injected with MOs at the 1-cell stage were evaluated for their phenotype at 55 hpf. (A) Control zebrafish embryo with magnified normal brain area. Scale bar = 500 µm. (B and C) z*bbs1* AUG MO-injected embryo and z*nphp7.2* SP1 MO-injected embryo with hydrocephalus. (D) The Tg(*WT1b:EGFP*) transgenic line shows normal pronephric glomerulus and tubules (Scale bar = 100 µm), (E and F) z*bbs1* AUG MO-injected embryo and z*nphp7.2* SP1 MO-injected embryo show pronephric cysts (asterisks). At 55 hpf, larval dysmorphy was categorized according to the visual scale (Fig. S3). The degree of dysmorphy was increased with the amount of injected MOs (G, z*bbs1* AUG MO; H, z*nphp7.2* SP1 MO). (G and H) Cystic pronephric phenotypes were dose-dependent in zebrafish embryos injected with z*bbs1* and z*nphp7.2* MO respectively. The graphs show percentages of the number (n) of embryos that were examined.

**Figure 4:**
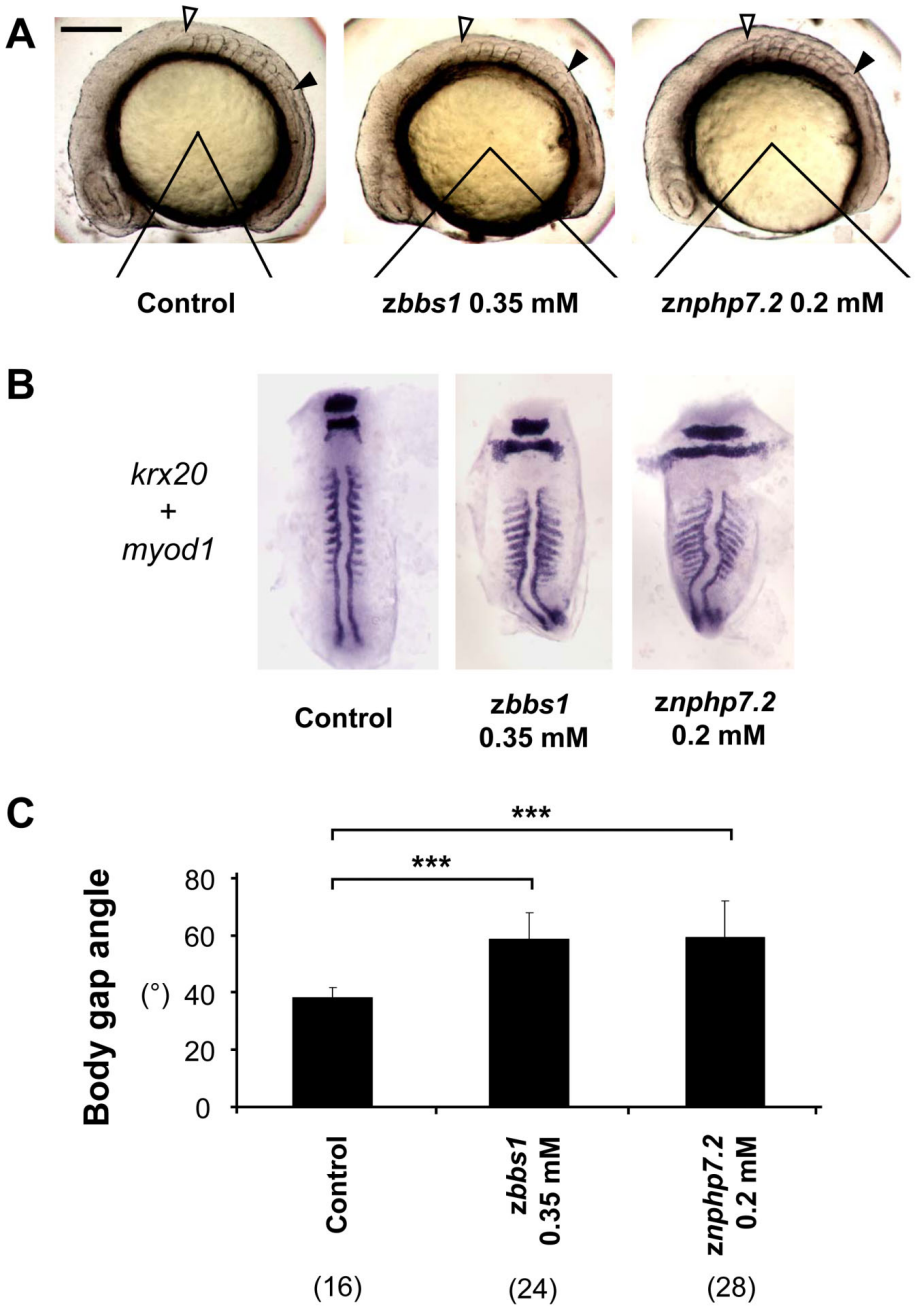
zBbs1 and zNphp7.2 morphant embryos showed defects in convergent extension movements. (A) Representative side views of control embryos and morphants demonstrate the analysis of the body gap angle which is inversely related to length of the body. Embryos were randomly picked at 8–10 somite stage. Open arrowhead marks first somite, filled arrowhead ninth somite respectively. Scale bar = 200 µm. (B) The expression of *krx20*/*myod1* in morphant embryos showed shortened body axis with significantly broader somites compared to control. (C) The morphants of z*bbs1* and z*nphp7.2* showed wider body gap angle compared to control embryos. The numbers in the brackets (n) are the numbers of total embryos which were examined.

**Figure 5:**
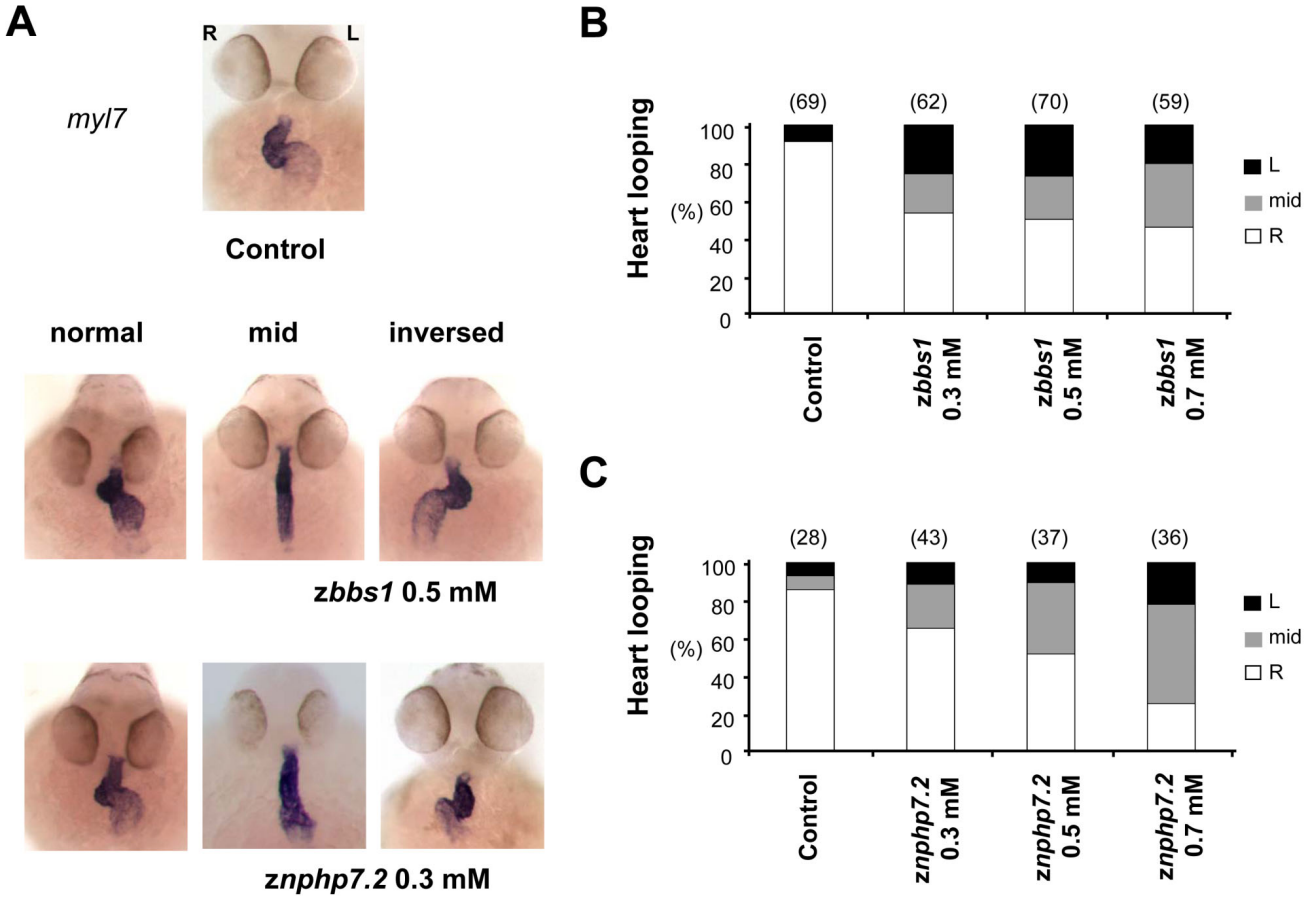
Depletion of zBbs1 and zNphp7.2 caused defects in heart looping. To examine organ laterality defects, zebrafish embryos were examined for changes in heart looping. (A) In situ hybridisation (*myosin light chain 7; myl7*) of both z*bbs1* and z*nphp7.2* morphant embryos at 55 hpf showed defective heart looping. (heart looping to the right (R = normal), without heart looping (mid) and looping to the left (L = inversed)) (B) Whereas the hearts of control embryos showed more than 90% rightward looping, approximately 50% of zBbs1-depleted embryos and (C) 35–75% of zNphp7.2-depleted embryos had *situs inversus* or mid position of the heart. The numbers in the brackets (n) are the numbers of total embryos which were examined.

To address the role of zBbs1 and zNphp7 in zebrafish kidney development, we performed knockdown experiments by injection of MO, targeting *zbbs1* translational start codon (AUG MO) and exon 2 splice donor site (SP MO) of *zbbs1* to deplete zBbs1 [33] (Fig. S2A). To test the efficacy of z*bbs1* AUG MO, we performed an *in vitro* transcription and translation (TNT) assay. This showed that z*bbs1* AUG MO efficiently interfered with zBbs1 protein expression (Fig. S2B). Both MOs showed similar phenotypes, which implies that the observed phenotypes were knockdown-specific. However, the AUG MO caused less general developmental side effects, and was therefore used in all subsequent experiments. To examine the effect of depletion of zBbs1 on kidney development *in vivo*, we used the transgenic fish line Tg(*wt1b:EGFP*) [34]. At 55 hpf, z*bbs1* morphants displayed cysts with distension of the neck segment of the pronephric tubule (Fig. 3E and G) indicating that zBbs1 is required for normal zebrafish pronephros development. The overall appearance of the morphant embryos was categorized by the level of dysmorphy ranging from wild-type-like (WT) to increasingly severe dysmorphy (D1 to D3) (Fig. 3G and Fig. S3). The grade of dysmorphy and the number of cystic morphants showed a MO dose dependency (Fig. 3G). In addition, we observed the previously described effects of zBbs1-depletion manifesting in hydrocephalus and curvature of the body axis (Fig. 3B and Fig. S3), in convergent extension (CE) defects (Fig. 4), and in defects of left-right (L-R) asymmetry, i.e. organ laterality, such as heart looping (Fig. 5A and B; Fig. S5A and B), and situs of liver and pancreas (Fig. S5C and D) [35], [36], [37]. Moreover, z*bbs1* deficient embryos showed defects in eye development displaying reduced eye size and impaired layer formation of the retina (Fig. S8) similar to the knockdown of the orthologue of retinitis pigmentosa gene 2 [38], [39]. Altogether, the phenotypes detected in z*bbs1* morphants were reminiscent of ciliopathy-associated phenotypic changes previously described in zebrafish [33], [35].

Depletion of zNphp7.1 by either translational initiation site blocking AUG MO, or by a MO binding to the exon 5 splice donor site did not result in any specific phenotype readily distinguishable from wild-type (data not shown). For knockdown of z*nphp7.2*, 2 different SP MOs, one targeting the exon 3 splice donor site (SP1) and the other targeting the exon 6 splice acceptor site (SP2) of z*nphp7.2* gene, were used (Fig. S4A). Both MOs caused similar phenotypes, confirming the specificity of the knockdown. However, the SP1 MO targeting exon 3 showed higher efficiency at lower concentrations and was used subsequently. *znphp7.2* MO injections in 1-cell embryos caused aberrant splicing of intron 3 in z*nphp7.2* pre-mRNA leading to 2 splice products both of which cause an early truncation within the first ZF domain eliminating all ZF domains together with the C-terminus of zNphp7.2 (Fig. S4B–D).

The phenotype of z*nphp7.2* depletion includes curvature of the body axis and hydrocephalus (Fig. 3), retinal degeneration with disturbed retinal layering and reduced eye size (Fig. S8) and cystic pronephros (Fig. 3C, F and H), which are very similar to the phenotype of z*bbs1* morphants pointing to defects in cilia function. Co-injection of *in vitro* transcribed z*bbs1* or z*nphp7.2* mRNA together with the corresponding MO partially rescued the morphant phenotype (Fig. S6), confirming the specificity of the MO-mediated knockdown.

To exclude that pronephric cyst formation was attributable to cloaca malformation, FITC-conjugated fluorescent dextran (70 kD) was injected into the common cardinal vein of control and morphant embryos with pronephric cysts at 96 hpf. The excretion of fluorescent dextran with the urine was observed shortly after the injection in 22 control embryos, 16 z*bbs1* and 21 z*nphp7.2* morphants (Fig. S7). Only, 2 z*nphp7.2* morphants showed cloaca blockage. Therefore, we conclude that pronephric cyst formation of z*bbs1* and z*nphp7.2* morphants was not caused by disruption of cloaca development (Fig. S7) [40].

Knockdown of z*nphp7.2* also caused CE defects judged by a wider body gap angle in relation to the stage of somitogenesis which translates into a shorter body axis and broader somites on in situ hybridization with probes for *krx20* and *myod1* (Fig. 4).

Cilia-driven fluid flow in the Kupffer's vesicle (KV) triggers symmetry breaking events during zebrafish development [37], [41], [42], [43], [44]. Defects in L-R asymmetry were observed in z*nphp7.2* morphant embryos for early laterality markers like *lefty2* (Fig. S5A and B) and in MO dose dependent manner for heart looping (Fig. 5A and C) and the situs of liver and pancreas (Fig. S5C and D). This shows the involvement of z*bbs1* and z*nphp7.2* in establishing organ laterality most likely via cilia function in KV.

### z*bbs1* and z*nphp7.2* show a genetic interaction in cystogenesis

**Figure 6:**
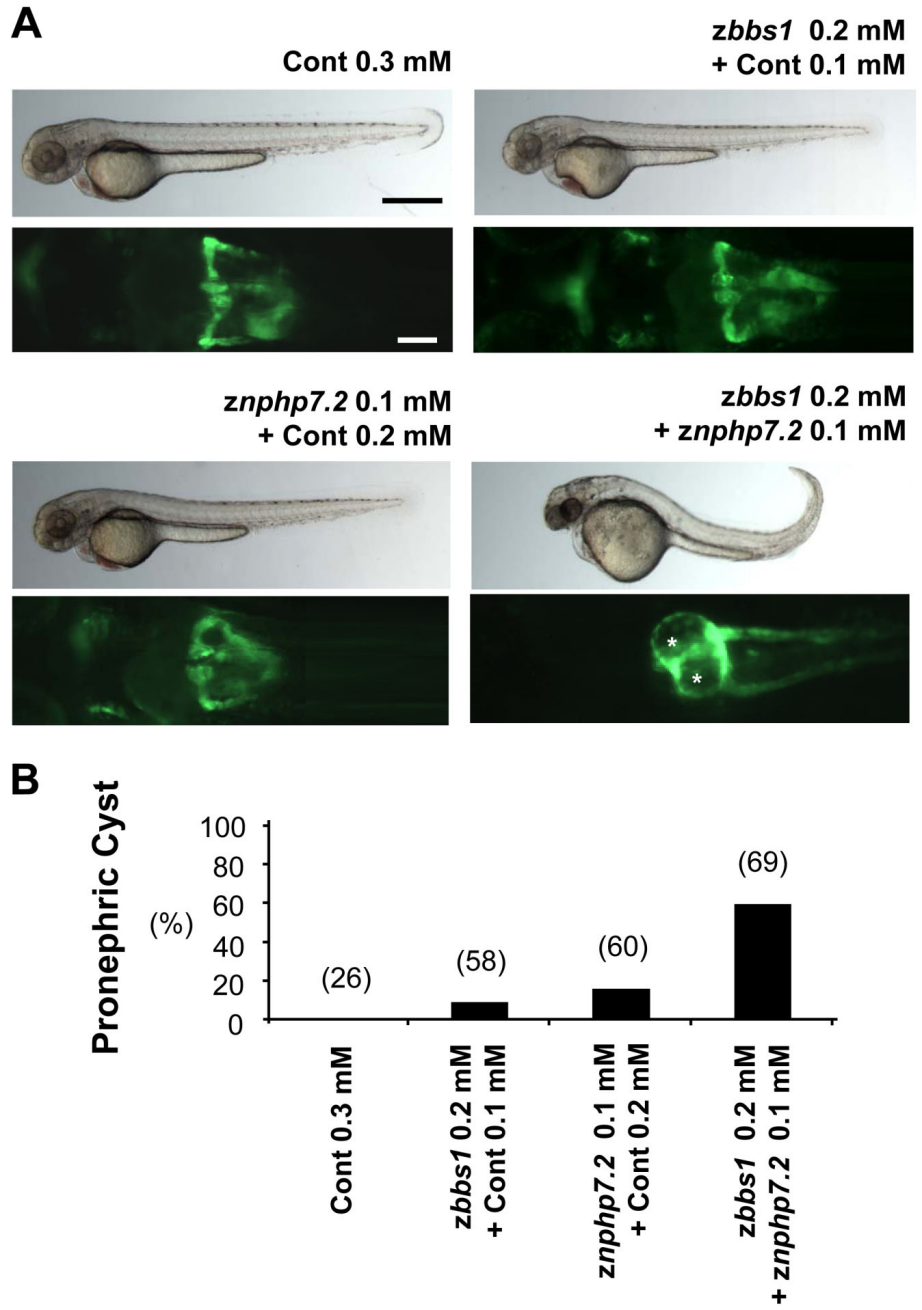
Genetic interaction between z*bbs1* and z*nphp7.2 in vivo*. Injected zebrafish embryos were assessed for the incidence of pronephric cysts. (A) Cont MO injected embryos with normal pronephros. While the majority of z*bbs1* or z*nphp7.2* morphants (suboptimal dose) showed pronephros of normal morphology, those of combined knockdown exhibited pronephros with cysts. Cysts are marked by asterisks. (Black scale bar = 500 µm, White scale bar = 100 µm) (B) Pronephric cysts were detectable after individual injections of *znphp7.2* MO at 0.1 mM and *zbbs1* MO at 0.2 mM with the level of 17% and 10% respectively whereas the combined knockdown caused pronephric cysts in 59% of microinjected embryos. The final MO concentration for injection was 0.3 mM in all conditions to keep total MO dose constant. Therefore suboptimal doses of z*bbs1* and z*nphp7.2* MO were combined with Cont MO to obtain this final concentration of 0.3 mM. The numbers in the brackets (n) are the numbers of total embryos which were examined.

The co-immunoprecipitation suggested a link between BBS1 and NPHP7 (Fig. 1). To confirm this interaction *in vivo*, we performed combined knockdown of z*bbs1* and z*nphp7.2* in zebrafish. Individual injections of a suboptimal dose of MO, z*bbs1* (z*bbs1* MO 0.2 mM+Cont MO 0.1 mM) and z*nphp7.2* (z*nphp7.2* MO 0.1 mM+Cont MO 0.2 mM), displayed 10% and 17% of pronephric cyst formation, respectively. However, combined knockdown of z*bbs1* and z*nphp7.2* (z*bbs1* MO 0.2 mM+z*nphp7.2* MO 0.1 mM) synergistically increased the number of embryos with cystic pronephros to 59%, suggesting a genetic interaction between z*bbs1* and z*nphp7.2* (Fig. 6). To keep the total amount of injected MO the same for all injections, the suboptimal doses of z*bbs1* and z*nphp7.2* were combined with Cont MO to achieve 0.3 mM as the final MO concentration for injection.

### Knockdown of z*bbs1* and z*nphp7.2* influences cilia motility

**Figure 7:**
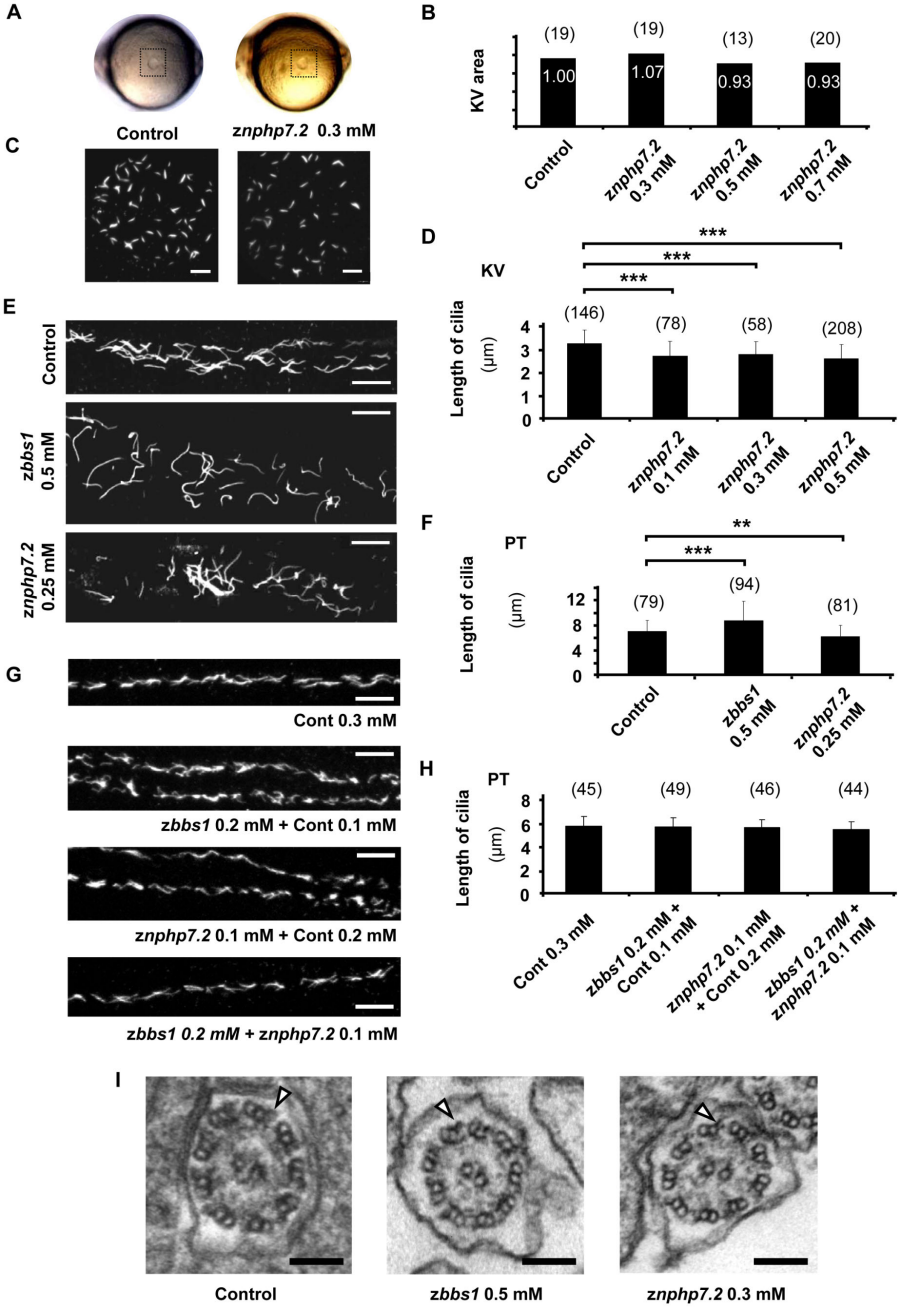
Knockdown of *znphp7.2* showed normal development of cilia. (A) Images of live zebrafish embryos at the 8–10 somite stage. Kupffer's vesicle is located in the dashed box. (B) Measurement of relative KV area with setting control as ‘1.00’. The knockdown of z*nphp7.2* did not significantly affect on KV development and area. (C) Images of cilia in the KV of 8–10 somite stage were stained for acetylated tubulin. Scale bar = 10 µm. (D) zNphp7.2-deficient embryos showed shortened length of cilia in KV compared to control embryos. (E) Staining of acetylated tubulin in the anterior pronephric tubule of morphants at 48 hpf displayed that the overall distribution of cilia remained unchanged compared to control (Scale bar = 10 µm) even though (F) the knockdown either of z*bbs1* showed longer and the knockdown of z*nphp7.2* showed shorter cilia. (PT, Pronephric Tubule) (G and H) The morphology and length of cilia in posterior pronephric tubule appeared unchanged in combined knockdown of z*bbs1* and z*nphp7.2* compared to single knockdown of z*bbs1* or z*nphp7.2* or compared to Cont MO injected embryos. (PT, Pronephric Tubule) (I) The morphants of z*bbs1* and z*nphp7.2* displayed normal ultrastructure of motile cilia in pronephric tubule without recognizable deficiency in dynein arms (outer dynein arm marked by arrowhead). The numbers (n) in the graphs are the number of total cilia which were examined. 4–6 individual embryos per group were examined.

As indicated above, knockdown of either z*bbs1* or z*nphp7.2* led to defects in cardiac looping in zebrafish embryos. One potential cause of this defect could be the defective “anlage” of the KV [45]. Indeed, our examinations revealed that depletion of zBbs1 causes defects in KV formation in a substantial number of embryos consistent with previous findings [33], while morphological examination of z*nphp7.2*-depleted zebrafish embryos showed normal formation of KV (Fig. 7A and B). Further examinations of the ciliogenesis in KV of z*nphp7.2* knockdown embryos were performed using immuno-staining for acetylated tubulin. This result indicated that development of cilia in the KV appeared shorter compared to control, however, the overall distribution of cilia in KV was unchanged (Fig. 7C and D). In addition, cilia formation in the pronephric tubule of zBbs1- and zNphp7.2-depleted embryos was not disturbed even though the cilia in zBbs1-depleted embryos were longer, and the cilia in zNPHP7.2-depleted embryos were shorter at significant level compared to control (Fig. 7E and F). The ciliary morphology and length in the pronephric tubule were also assessed in the combined knockdown condition of both z*bbs1* and z*nphp7.2* which showed the synergistic enhancement of cystic pronephros phenotype. In this condition, the experimental results showed that single and simultaneous knockdown of z*bbs1* and z*nphp7.2* does not affect cilia length in pronephric tubule (Fig. 7G and H). This data raised the question, whether the motility of cilia in morphant zebrafish embryos was impaired. Therefore, we assessed cilia motility by live embryo confocal imaging using the Tg(*βact:Arl13b–GFP*) transgenic line which labels cilia with GFP in various organs [46]. We used this fish line to be able to detect subtle changes in motility within the population of cilia of one tubule. The movies were obtained along the whole pronephric tubule. For better comparison, three regions were defined as anterior, middle, and posterior (Fig. S9A). Regular beating movements of cilia in the lumen of the pronephric tubule in downstream direction, towards the cloaca, were observed in control zebrafish embryos (Movie S1, S2 and S3) as described previously [37]. The movies were recorded from morphant embryos with clear pronephric cysts. Strikingly, the cilia in z*bbs1* (0.5 mM)- and z*nphp7.2* (0.2 mM)-depleted zebrafish embryos exhibited greatly reduced motility and abnormal beating pattern (Movie S4 and S5, S6 for z*bbs1* MO; Movie S7 and S8, S9 for z*nphp7.2* MO). These results suggest that zBbs1 and zNphp7.2 are required for motility of cilia in pronephric tubule of zebrafish.

Furthermore, disturbed cilia movement was detected in the nasal pit of z*bbs1* and z*nphp7.2* morphants at 72 hpf, confirming that zBbs1 and zNphp7.2 play a role in cilia motility. This was monitored by ordinary videomicroscopy with a high-speed camera system (Movie S10, S11 and S12). Immuno-staining of cilia in the nasal pit with acetylated tubulin antibody shows that formation of cilia in the nasal pit is unimpaired in z*bbs1* and z*nphp7.2* morphant embryos (Fig. S9B).

To scrutinize the cause of the impaired motility of cilia, electron microscopy was performed to examine ultrastructure of cilia in the pronephric tubule of z*bbs1* and z*nphp7.2* morphants: ultrastructural defects, e.g. deficiency of dynein arms, could not be detected in pronephric cilia of both morphant embryos (Fig. 7I). Hence, we conclude that the disturbed motility of z*bbs1* and z*nphp7.2* morphant cilia is not necessarily associated with the axonemal ultrastructure of cilia [47].

To examine the interaction between z*bbs1* and z*nphp7.2*, cilia beating in the pronephric tubule of morphants with combined knockdown condition was analyzed. Interestingly, the simultaneously zBbs1- and zNphp7.2-depleted embryos displayed severely impaired motility of cilia compared to zBbs1- or zNphp7.2-deficient embryos (Movie S13, S14, S15, S16, S17, S18, S19, S20, S21, S22). In the movies of combined knockdown embryos, the arrows point to the individual cilia showing impaired movement. The movies were recorded from the single knockdown morphant embryos without pronephric cysts, whereas the movies were recorded from the combined injected morphant embryos showing clear pronephric cysts. This result supports the genetic interaction between z*bbs1* and z*nphp7.2* which was shown for pronephric cystogenesis in this study.

Therefore, we conclude that the cystic pronephros phenotype in the knockdown of z*bbs1* and z*nphp7.2* is due to the aberrant motility of cilia and disturbed fluid flow in the pronephric tubule [37], [48].

## Discussion

Multiple ciliopathy-associated genes have been identified that are involved in syndromic cystic kidney disease. Despite phenotypic overlap, the functional relationship and interactions are not well understood yet [4]. The clinical features and the severity of phenotypic changes vary with different identified mutations within one gene, but also with the mutational load and the combination of mutations in two or more genes of ciliopathy-associated gene families [49], [50], [51], [52]. Based on the clinical overlap, we speculated that the similarity in phenotypes is reflected by functional interaction(s) between BBS and NPH gene products. Immunoprecipitation assays identified an interaction between BBS1 and NPHP7. The interacting domain was located to the N-terminus of NPHP7. To examine the *in vivo* relevance of this interaction, we identified the zebrafish homologues of NPHP7, zNphp7.1 and zNphp7.2, likely the result of zebrafish genome duplication events [53]. The protein sequence identity between zebrafish and human is high within the zinc finger region as expected for the GLI/GLIS protein families (human sequence aa168–317, http://smart.embl-heidelberg.de) [27], [28]. Since the knockdown of z*nphp7.1* was phenotypically identical to control embryos, the biological function of *znphp7.1* remains unclear. The phenotypic changes (curved body axis, hydrocephalus, retinal degeneration and pronephric cyst formation) induced by knockdown of z*nphp7.2* were compatible with depletion of other ciliopathy gene products [37]. We excluded cyst formation on the grounds of cloaca malformation by fluorescent dye injection und urinary excretion experiments. There is no detailed description of the clinical phenotype available in the Canadian Oji-Cree kindred with the first known human *NPHP7/GLIS2* mutation (homozygous splice-site mutation, c.755+1G>T) and recently found second mutation in the Turkish patient (c.523T>C, p.C175R) [29], [54]. In zebrafish, knockdown of z*nphp7.2* induced defects in left-right asymmetry of organ situs consistent with the concept that mutations of NPHP7 belong to the growing family of ciliopathies. Kupffer's vesicle revealed a normal size and shape in z*nphp7* MO-injected zebrafish embryos, whereas defects in formation of Kupffer's vesicles were described in z*bbs1* MO-injected zebrafish embryos [33], suggesting that the laterality defects in z*nphp7* morphant embryos were due to a ciliary or post-ciliary event. Both zBbs1- and zNphp7.2-depleted embryos displayed defects in CE movements, previously described for the knockdown of z*bbs1* [36]. Knockdown of z*bbs1* expands the expression of *axin2*, a downstream target of canonical Wnt signalling [36], and Glis2 negatively modulates Wnt/beta-catenin signalling [55]. Thus, both zBbs1 and zNphp7 appear to facilitate non-canonical Wnt while antagonizing canonical Wnt signalling. The synergistic effect of *znphp7.2* and *zbbs1* MOs on cyst formation further supports the notion that *BBS1* and *NPHP7* (*GLIS2*) act within the same or overlapping signalling pathways.

The phenotypic changes observed in either *znphp7*- or *zbbs1*-depleted zebrafish embryos clearly suggested a defect of motile cilia or cilia connected signalling pathways. We first excluded defects in ciliary axoneme formation: Although the ciliary length, measured in the Kupffer's vesicle or pronephric tubule, was significantly different in z*nphp7.2*- and z*bbs1*-depleted zebrafish embryos compared to control, there was no change in ciliary length observed in the set of experiments with combined MO injection. Furthermore, the general distribution and electron microscopic ultrastructure revealed no difference between morphant and control. However, our results of video-microscopy and confocal microscopy revealed an astonishingly impaired ciliary motility in pronephric tubule and nasal pit of z*nphp7.2* and z*bbs1* morphants, suggesting that the ciliopathy-associated phenotypes caused by the knockdown of either z*bbs1* or z*nphp7.2* are due to the disruption of ciliary motility [37].

In primary cilia dyskinesia (PCD) the impaired ciliary motility often goes along with changes in ultrastructure, e.g. deficiency of dynein arms, central pair complex or radial spokes, but there have been reports of unchanged ciliary ultrastructure with DNAH11 [56]. With DNAH11 one possible explanation is that the mutant DNAH11 protein retains its N-terminal domain, which is required for the correct incorporation of outer dynein arms [57]. Another key mechanism for regulation of ciliary motility is phosphorylation, i.e. phosphorylation/dephosphorylation of the dynein arms, through kinases and phosphatases that are anchored immediately adjacent to their axonemal substrates [58]. These kinases and phosphatases could be candidates regulated by events downstream of z*nphp7.2* and z*bbs1* without causing ultrastructural defects on electron microscopic level.

Previous reports localized BBS1 and GLIS2 to cilia [23], [29], [31]; this is particularly remarkable for the transcription factor NPHP7/GLIS2 preferably found in the cell nucleus. Recently, the BBSome, structurally related to coat complexes, has been reported to mediate the transport of cargo to the ciliary compartment [23]. BBS1 interacts with other proteins such as BBS3, LepRb, Rabin8, and has been implicated in protein and membrane trafficking to cilia [10], [59], [60]. Thus, BBS1 might be involved in the transport of GLIS2 to the cilium, where posttranslational modifications of GLIS2 could take place similar to the mandatory processing of GLI transcription factors in the cilium [61], [62]. Cleavage of GLIS2 has been described to be induced for example by p120 catenin [63]. Alternatively BBS1 could also facilitate the transport of GLIS2 from the cytosol to the nucleus. Curiously, other NPHP gene products such as NPHP2/Inversin [64] and NPHP15/CEP164 [65] have been shown to localize to both the cilium as well as the nucleus.

In conclusion, we demonstrate that depletion of Nphp7/Glis2 leads to a NPH-typical phenotype in zebrafish including defects in left-right organ asymmetry, further validating Glis2 as a legitimate member of the NPH gene family in spite of the limited description of human mutations. We also show that Bbs1 and Nphp7 are both required for normal ciliary motility, a pathway responsible for normal body asymmetry. Although the presence of BBS1/NPHP7 in the cilium suggests a cilia-specific function, this module could also regulate the expression of other gene products required for ciliary motility.

## Methods

### Zebrafish lines

Wild-type TL or TÜAB zebrafish were maintained and raised as described (Westerfield, 1995). Dechorionated embryos were kept at 28.5°C in E3 solution with or without 0.003% 1-Phenyl-2-thiourea (PTU, Sigma) to suppress pigmentation and staged according to somite number (som) or hours post-fertilization (hpf) (Westerfield, 1995).

Westerfield M: The Zebrafish Book. A Guide for the Laboratory Use of Zebrafish (Danio rerio), University of Oregon Press 1995.

The transgenic fish line Tg(*wt1b:eGFP*) was a kind gift of Christoph Englert [34]. The Tg(*βact:Arl13b–GFP*) transgenic line we received thankworthy from Brian Ciruna's lab [46].

### Zebrafish embryo manipulations

Embryos at 1-cell stage were microinjected with 4 nl of solution containing MO (MO; Gene Tools LLC) or *in vitro* transcribed capped mRNA, diluted in 100 mM KCL, 0.1% Phenol Red and 10 mM HEPES, pH 7.5. 2 different MOs targeting zebrafish *bbs1* were used, a translation-blocking MO (z*bbs1* AUG MO; 5′-GGGAACAGATGACATGGTTGTTTTG-3′) and an MO targeting against the splice donor site of exon 2 (z*bbs1* Ex2 MO; 5′-TGAAACTCACCAATGCATGAGGAGA-3′) [33]. We designed the 2 independent MOs against z*nphp7.1* (XM_001340674.2 which was identified in Ensembl Zv7), targeted to translation initiation site (z*nphp7.1* AUG MO; 5′-GCTCGTCCAGTGACAGCATGGCTTT-3′) and exon 5 splice donor site (z*nphp7.1* SP MO; 5′-TATAATATCCACAGTCTGACCTGGC-3′). 2 separate MOs against z*nphp7.2* were also designed and ordered, targeted to exon 3 splice donor site (z*nphp7.2* SP1 MO; 5′-TATCCCTTACTCAATCTCACCTTCC-3′) and exon 6 splice acceptor site (z*nphp7.2* Ex6 MO; 5′-CTCACCTAAAACACAAGTACAGAGA-3′). In order to decrease the nonspecific effects of the reagents to zebrafish embryos, all MOs were co-injected with p53 MO (0.1 mM). p53 MO (0.1 mM) was also injected into all wild-type embryos as controls [66]. For combined knockdown experiment with 2 different MOs simultaneously, all MO injection samples were balanced with Cont MO (standard control MO, Gene Tools LLC.) MO to have 0.3 mM in order to inject the same amount of MOs in total. The clone of zebrafish *bbs1* homologue was obtained from ImaGenes GmbH. The full-length z*bbs1* was isolated by PCR and cloned into the vector pCS2+ for *in vitro* transcription/translation assay. To produce the z*bbs1* mRNA for rescue experiment, we modified the sequence of z*bbs1* with sequence-directed mutagenesis and generate a construct with 3 silent mutations in z*bbs1* AUG MO binding site (original sequence from start codon, atg tca tct gtt; modified sequence, atg tct tca gta). The full-length of z*nphp7.2* obtained by PCR using zebrafish embryonic cDNA was also inserted into pCS2+ (*znphp7.1* (Genbank KF054060, Zv9: ENSDARG00000078388) and *znphp7.2* (Genbank KF054061, Zv9: ENSDARG00000073861)). Phenotype rescue experiments were performed with *in vitro* transcribed 20 pg of full-length MO-resistant z*bbs1* mRNA or 10 pg of wild-type z*nphp7.2* mRNA (mMessage Machine kit, Ambion) which was co-injected with z*bbs1* AUG MO (0.4 mM) or z*nphp7.2* SP1(0.3 mM) respectively.

The body gap angle of embryos at 8–10 somite stage was measured to assess convergent extension movement [36]. KV areas of control and morphant embryos were measured at 8–10 somite stage using imageJ (http://rsb.info.nih.gov/ij/). The average area measured in control embryos was set as 1.00 to determine other measurement of morphants.

All animal studies were approved by the Committee on Research Animal Care, Regierungspräsidium Freiburg (35-9185.81/G-11/41).

### Cell culture and transfection

For co-immunoprecipitations, HEK 293T cells (received from American Type Culture Collection, ATCC, Manassas, VA) were grown in DMEM with 10% FBS. The 70–90% confluent cells were split at a ratio of 1∶5 and transfected the next day with plasmid DNA applying the calcium phosphate method as described previously [67]. The transfection was stopped after 6–8 h by replacing the media.

### Co-immunoprecipitation

To construct Flag-tagged BBS1, NPHP7 and truncations of NPHP7/GLIS2, the cDNAs of full-length *BBS1* and *NPHP7* were amplified from the EST clone by PCR and cloned between MluI and NotI site of the Flag-pcDNA6. HEK (Human embryonic kidney) 293T cells were transiently transfected with plasmids by the calcium phosphate method, and lysed after incubation for 24 h in immunoprecipitation buffer containing 20 mM Tris-HCl (pH 7.5), 1% Triton-X 100, 25 mM NaF, 12.5 mM Na4P2O7, 0.1 mM EDTA, 50 mM NaCl, 2 mM Na3VO4, and protease inhibitors. After lysates were cleared by centrifugation at 15 000 g for 30 min at 4°C, the supernatants were incubated with 30 µl of anti-Flag-M2 beads for 3 h [68]. The beads were washed extensively with immunoprecipitation buffer, and the retained proteins were analyzed with western blot analysis. Antibodies used in this western blot analysis, included FLAG® monoclonal antibody produced in rabbit (Sigma, Hamburg, Germany), rabbit antibody to V5 (Serotec).

### Eye size measurement and HE Staining for eye sections

Eye size measurement and HE Staining for eye sections were performed with 80 hpf and 96 hpf embryos, respectively. Image J (http://rsb.info.nih.gov/ij/) was used for measurement of the area of the eye. The embryos at 96 hpf that were sectioned for Hematoxylin and Eosin staining were fixed in BT-fix (4% PFA, 0.1 M Na_2_HPO_4_ buffer (pH 7.3), 3% sucrose, 0.12 mM Ca_2_Cl) at 4°C overnight. After being washed in PBS and taken through an ethanol dehydration series, the embryos were embedded in JB-4 resin (Polyscience Inc.) and sectioned at 3–5 µm using a microtome (Leica). Slides were stained for Hematoxylin and Eosin.

### In situ hybridisation and immunohistochemistry

In situ Hybridisation (ISH) was carried out as described previously [69]. Dig-labelled antisense RNA was transcribed with T3 or T7 RNA polymerase (Roche). The *myl7* (*myosin, light polypeptide 7*) antisense probes were generated by linearizing template plasmids with NotI and transcription with T7 RNA polymerase. *krx20* (*krox20*) antisense probe was produced by linearizing template plasmids (pBS KS vector) with PstI and transcription with T3 RNA polymerase. *myod1* (*myogenic differentiation 1*) in pCRII-Topo was linearized by BamHI and transcribed by T7. *lft2* (*lefty2*) in pCS2+ was linearized by ClaI and transcribed by T7. *ins* (*preproinsulin*) and *foxa3* (*forkhead box A3*) probes in pBS SK vector were linearized with XbaI and XhoI respectively and transcribed with T7 and T3 RNA polymerase respectively.

For immunohistochemistry, primary mouse monoclonal antibodies, anti-acetylated tubulin (Sigma) were used in a 1∶500 dilution. Secondary anti-mouse antibodies labeled with Cy3 (Jackson ImmunoResearch and Molecular Probes) were used. Embryos were fixed in 1% DMSO and 4% PFA in PBS and stained with primary and secondary antibodies by conventional whole-mount antibody staining method. The length of stained cilia was measured with imageJ.

### RT PCR analysis

The total RNA was extracted from entire zebrafish embryos at desired stage or from adult zebrafish organs (kidney, eye, heart, testis, gut and muscle) with RNeasy® MiniKit (Qiagen). cDNA was synthesized from the total RNA using ProtoScript® First Strand cDNA Synthesis Kit (Biolabs Inc.). These cDNAs were utilized for further PCR analyses. zebrafish *ef1α* (*translation elongation factor 1 alpha*) was amplified as a positive control. The following primers were used for corresponding PCR analysis: z*bbs1* (forward: 5′-tgttccccaggagttgaaag-3′; reverse: 5′-gtaacaggggctggcaaata-3′), z*nphp7.1* (forward: 5′-gttcttcctgccgattggtg-3′; reverse: 5′-tcattggtatgagtgcggat-3′), z*nphp7.2* (forward: 5′-tgctccccagagctctccaa-3′; reverse: 5′-ggcaacagtagccagaatccttc-3′), z*ef1α* (forward: 5′-atctacaaatgcggtggaat-3′; reverse: 5′-ataccagcctcaaactcacc-3′)

### Fluorescent dextran injection

For urine excretion assays, 5% FITC-conjugated dextran solution (70 kD) (Molecular Probes) was injected into the common cardinal vein of 96 hpf embryos anesthetized with 0.2 mg/ml tricaine (3-aminobenzoic acid ethylester, Sigma) in egg water.

### Transmission electron microscopy

Zebrafish embryos at 55 hpf were fixed with PBS containing 1% PFA/2% glutaraldehyde for 1 h at RT and post-fixed with 1% osmium tetroxide (Polyscience) for 30 min at RT. The embryos were dehydrated in ethanol (50% and 60% for 10 min each) and transferred into 1% uranyl acetate (Polyscience) in 70% ethanol overnight at 4°C.

The embryos were subjected to further dehydration through a series of 80%, 90%, 98% and 100% ethanol. After washing the embryos with propylene oxide, they were embedded in Durcupan (Fluka). Ultra-thin sectioning was performed with a Leica EM UC6. Serial sagittal sections (60 nm) were conducted from rostral to caudal (from the beginning of the yolk extensions to copper-grids). Sections were observed and imaged in an electron microscope (LEO 906E; Carl Zeiss). All images were exported as TIFF files.

### Confocal microscopy with Tg(*βact:Arl13b–GFP*) transgenic line

All movies were taken with LSM-I-Live Duo ZEISS LSM 510 DUO using ZEN 2010 software (Carl Zeiss, Inc.) and laser diode 489. Image acquisition for the moving cilia was 30 frames per second and the recorded movies are replayed in real-time. The movement of cilia in pronephric tubule was imaged with 55 hpf embryos of the Tg(*βact:Arl13b–GFP*) transgenic line [43]. The embryos were incubated with 40 mM BDM (2,3-butanedione monoxime, Sigma-Aldrich) for 5 min to eliminate the interfering signal of blood flow, and then the mounted embryos in 1% low melting temperature agarose were covered with egg water (20 mM BDM) for imaging. The movies were taken with LD LCI Plan-Apochromat 25×/0.8 (zoom: 2.0).

### High-speed videomicroscopy

The 55 hpf phenylthiourea (PTU)-treated embryos were put in E3 egg water containing tricaine. The imaging of beating cilia in the nasal pit was conducted on a Zeiss Axioplan microscope (Carl Zeiss, Jena, Germany) using a 63×/0.55 water immersion lens installed with a high-speed Photron FastCAM-PCI 500 videocamera (Photron LTD) [37]. Image acquisition was 250 frames per second by Photron FastCAM version 1.2.0.7 (Photron LTD). The movies are replayed in 30 frames per second.

### Protein blotting and *in vitro* translation

1 µg of plasmids DNA (wild-type full-length z*bbs1* in pCS2+) were mixed with or without MOs and *in vitro* transcription/translation reactions were performed in the presence of biotinylated lysine according to manufacturer's manual (TNT® Quick Coupled Transcription/Translation Systems, Transcend™ Non-Radioactive Translation Detection Systems, Promega). 1 µl of the reaction was diluted in 14 µl of 1× protein sample loading buffer. The reaction mixture was analyzed by SDS-PAGE and protein blotting using HRP-conjugated streptavidin.

### Statistical analyses

Statistical analyses, Mann-Whitney rank sum test and Student's t-test, were performed with SigmaStat® (Systat Software Inc.). Student's t-test was used for the data of ciliary length, eye size and KV area measurement. In the case of the comparison of body gap angle, t-test could not be applied. Therefore, the differences in the variance between control- and morphant-embryo body gap angle were analyzed using the Mann-Whitney Rank Sum Test. The probability value of P<0.05 was considered statistically significant (***, P≤0.001; **, P≤0.01; *, P≤0.05). *X^2^* (chi-square) test was performed for the result of morphant rescue experiments: Degrees of freedom = 1, alpha level of significance = 0.05 (College of Saint Benedict & Saint John's University) (http://www.physics.csbsju.edu/stats/contingency_NROW_NCOLUMN_form.html)

## Supporting Information

Figure S1 **Alignment of the amino acid sequences of the 2 variants of maternally expressed z** ***nphp7.2*** **transcripts.** The alignment shows that 1 amino acid is substituted (S99R) and 18 amino acids are deleted (aa 101–118) in maternally expressed transcript variant 2 (Transcript 2) compared to transcript variant 1 (Transcript 1) of z*nphp7.2*.
(TIF)

Figure S2 **Antisense morpholino oligonucleotides against z** ***bbs1*** **.** (A) MO (red line) targeting z*bbs1* translational start codon (AUG MO) and MO targeting exon 2 splice donor site (SP MO) of z*bbs1*. (B) 1 µg of plasmids DNA containing wild-type full-length z*bbs1* in pCS2+ vector was mixed with or without MOs *in vitro* translation reactions. z*bbs1* AUG MO efficiently interfered with zBbs1 protein expression.
(TIF)

Figure S3 **Grading of dysmorphy.** (A) Embryos at 48–55 hpf were assessed and scored as wild-type-like or with a degree of dysmorphy ranging from mild (D1) to severe (D3). (B) z*bbs1* or z*nphp7.2* morphant embryos were always consist of a mixed population of individuals with dorsally or ventrally curved body axis.
(TIF)

Figure S4 **Antisense morpholino oligonucleotides against z** ***nphp7.2*** **.** (A) 2 independent MOs against exon 3 donor site (SP1 MO) and exon 6 acceptor site (SP2 MO) of z*nphp7.2* were designed. (B and C) RT-PCR was performed with z*nphp7.2* SP1 MO-injected embryos at 55 hpf. The following PCR with the primers (short blue lines) designed to produce 325 bp amplicon of coding sequence of wild-type z*nphp7.2* cDNA was performed. z*nphp7.2* SP1 MO efficiently interfered with normal splicing to cause the insertion of intron sequence between exon 3 and exon 4 resulting in 2 abnormally large amplicons. (Exon 3 is marked as green to visualize the mRNA splicing process.) (D) These abnormal splicing products have a stop codon in the intronic sequence leading to a truncation within the first ZF domain after translating 6 extra amino acids from intronic sequence. This eliminates all other ZF domains together with the C-terminus of zNphp7.2.
(TIF)

Figure S5 **zBbs1- or zNphp7.2-deficient embryos showed defects in organ laterality.** (A, B) In situ hybridisation of both z*bbs1* and z*nphp7.2* morphants at 24 hpf with *lefty2* probe showed defective left-right asymmetry patterning. (C and D) Defective laterality of liver (*foxa3*) and pancreas (*ins*) was observed in zBbs1- and zNphp7.2-depleted embryos at 48 hpf.
(TIF)

Figure S6 **Co-Injection of z** ***bbs1*** **or z** ***nphp7.2*** **mRNA partially rescued the corresponding morphant phenotype.** Co-injection of 20 pg of z*bbs1* mRNA (A and B) or 10 pg of z*nphp7.2* mRNA (C and D) together with the corresponding MO partially rescued the dysmorphic changes caused by the MO-mediated knockdown, decreasing the dorsal body curvature and rescued pronephric cyst formation. (A: scale bar = 500 µm, B: scale bar = 100 µm) (D, *X^2^* = 5.27, P = 0.022; H, *X^2^* = 14.5, P<0.001).
(TIF)

Figure S7 **Dextran injection revealed fluid excretion via the cloaca in z** ***bbs1*** **and z** ***nphp7.2*** **morphants.** Zebrafish control and morphant embryos with pronephric cysts at 96 hpf were injected with 5% FITC-conjugated dextran solution (70 kD) into the circulation. Fluorescent dye excretion with the urine at the cloaca (black and white arrows) was observed in control embryos (22/22), z*bbs1* (16/16) and z*nphp7.2* (21/23) morphants. The lower panel represents a z*nphp7.2* morphant embryo with missing fluorescent dye excretion due to persistent closure of the cloaca (arrowheads). Images on the left column represent transmitted light images and images on the right column represent fluorescent images of the same embryo for each setting.
(TIF)

Figure S8 **z** ***bbs1*** **and z** ***nphp7.2*** **morphants display reduced eye size and defective retinal layer formation.** The eye size as area (µm^2^) was measured for control embryos, z*bbs1* and z*nphp7.2* morphant embryos at 80 hpf. (A) Representative brightfield images showing reduced eye size for *zbbs1* and z*nphp7.2* morphants in comparison to the control (Scale bar = 200 µm). (B) Statistical quantification of the measurements proved that the reduction in eye size for z*bbs1* and z*nphp7.2* morphants was significant in comparison to the control. (C) Histological cross-sections of 96 hpf z*bbs1* and z*nphp7.2* morphants revealed defective layer formation in comparison to the control. Two representative images are shown for each setting. (Scale bar = 100 µm).
(TIF)

Figure S9 **The morphants of z** ***bbs1*** **and z** ***nphp7.2*** **exhibited impaired motility of cilia in the pronephric tubule.** (A) The areas where the movies were recorded are shown by the dashed box. (B) Acetylated tubulin staining demonstrated normal development of cilia in the nasal pit (Scale bar = 10 µm). 3-dimensional (3D) images (upper panel) and z-plane images (lower panel) show that the cilia formation in the morphants of z*bbs1* and z*nphp7.2* is normal compared to control embryo.
(TIF)

Movie S1 **The anterior part of pronephric tubule in a control embryo.** The pronephric cilia of control embryo show normal motility.
(AVI)

Movie S2 **The posterior part of pronephric tubule in a control embryo.** The pronephric cilia of control embryo show normal motility.
(AVI)

Movie S3 **The cloaca area of a control embryo.** The pronephric cilia of control embryo show normal motility.
(AVI)

Movie S4 **The anterior part of pronephric tubule in a z** ***bbs1*** **morphant embryo.** The morphant embryo (z*bbs1* 0.5 mM) exhibited greatly reduced or abnormal motility. The arrows point to the individual cilia showing impaired movement. The movies were recorded from morphant embryos with clear pronephric cysts.
(AVI)

Movie S5 **The middle part of pronephric tubule in a z** ***bbs1*** **morphant embryo.** Most of the cilia in a morphant embryo (z*bbs1* 0.5 mM) exhibited greatly reduced or abnormal motility. The movies were recorded from morphant embryos with clear pronephric cysts.
(AVI)

Movie S6 **The cloaca area of a z** ***bbs1*** **morphant embryo.** Most of the cilia in a morphant embryo (z*bbs1* 0.5 mM) exhibited greatly reduced or abnormal motility. The movies were recorded from morphant embryos with clear pronephric cysts.
(AVI)

Movie S7 **The anterior part of pronephric tubule in a z** ***nphp7.2*** **morphant embryo.** Most of the cilia in a morphant embryo (z*nphp7.2* 0.2 mM) exhibited greatly reduced or abnormal motility. The movies were recorded from morphant embryos with clear pronephric cysts.
(AVI)

Movie S8 **Another anterior part of pronephric tubule in a z** ***nphp7.2*** **morphant embryo.** Most of the cilia in a morphant embryo (z*nphp7.2* 0.2 mM) exhibited greatly reduced or abnormal motility. The movies were recorded from morphant embryos with clear pronephric cysts.
(AVI)

Movie S9 **The cloaca area of a z** ***nphp7.2*** **morphant embryo.** Most of the cilia in a morphant embryo (z*nphp7.2* 0.2 mM) exhibited greatly reduced or abnormal motility. The movies were recorded from morphant embryos with clear pronephric cysts.
(AVI)

Movie S10 **The cilia in nasal pit of a control embryo.** The cilia in nasal pit of control embryo showed normal uniformed beating along the whole epithelial layer. Arrows indicate the border of the nasal pit.
(AVI)

Movie S11 **The cilia in nasal pit of a z** ***bbs1*** **morphant embryo.** The morphant embryo (z*bbs1* 0.4 mM) exhibited remarkably impaired motility with preserved motility only in focal areas. Arrows indicate the border of the nasal pit.
(AVI)

Movie S12 **The cilia in nasal pit of a z** ***nphp7.2*** **morphant embryo.** The morphant embryo (z*nphp7.2* 0.2 mM) exhibited remarkably impaired motility with preserved motility only in focal areas. Arrows indicate the border of the nasal pit.
(AVI)

Movie S13 **The middle part of pronephric tubule in a control morphant embryo.** The pronephric cilia of control morphant embryo (Cont MO 0.3 mM) show normal motility.
(AVI)

Movie S14 **The posterior part of pronephric tubule in a control morphant embryo.** The pronephric cilia of control morphant embryo (Cont MO 0.3 mM) show normal motility.
(AVI)

Movie S15 **The cloaca area of a control morphant embryo.** The pronephric cilia of control morphant embryo (Cont MO 0.3 mM) show normal motility.
(AVI)

Movie S16 **The middle part of pronephric tubule in a z** ***bbs1*** **morphant embryo.** The pronephric cilia of z*bbs1* morphant embryo (z*bbs1* 0.2 mM+Cont MO 0.1 mM) show active motility. The movies were recorded from the single knockdown morphant embryos without pronephric cysts.
(AVI)

Movie S17 **The cloaca area of a z** ***bbs1*** **morphant embryo.** The pronephric cilia of z*bbs1* morphant embryo (z*bbs1* 0.2 mM+Cont MO 0.1 mM) show active motility. The movies were recorded from the single knockdown morphant embryos without pronephric cysts.
(AVI)

Movie S18 **The middle part of pronephric tubule in a z** ***nphp7.2*** **morphant embryo.** The pronephric cilia of z*nphp7.2* morphant embryo (z*nphp7.2* 0.1 mM+Cont MO 0.2 mM) show active motility. The movies were recorded from the single knockdown morphant embryos without pronephric cysts.
(AVI)

Movie S19 **The posterior part of pronephric tubule in a z** ***nphp7.2*** **morphant embryo.** The pronephric cilia of z*nphp7.2* morphant embryo (z*nphp7.2* 0.1 mM+Cont MO 0.2 mM) show active motility. The movies were recorded from the single knockdown morphant embryos without pronephric cysts.
(AVI)

Movie S20 **The anterior part of pronephric tubule in a combined morphant embryo.** The pronephric cilia of morphant embryos with combined knockdown (z*bbs1* 0.1 mM+z*nphp7.2* 0.2 mM) showed more severe defective movements compared to z*bbs1* or z*nphp7.2* single knockdown morphants (Movie S16, S17, S18, S19). The movies were recorded from the combined injected morphant embryos showing clear pronephric cysts.
(AVI)

Movie S21 **The posterior part of pronephric tubule in a combined morphant embryo.** The pronephric cilia of morphant embryos with combined knockdown (z*bbs1* 0.1 mM+z*nphp7.2* 0.2 mM) showed more severe defective movements compared to z*bbs1* or z*nphp7.2* single knockdown morphants (Movie S16, S17, S18, S19). The movies were recorded from the combined injected morphant embryos showing clear pronephric cysts. The arrows point to the individual cilia showing impaired movement.
(AVI)

Movie S22 **The cloaca area of a combined morphant embryo.** The pronephric cilia of morphant embryos with combined knockdown (z*bbs1* 0.1 mM+z*nphp7.2* 0.2 mM) showed more severe defective movements compared to z*bbs1* or z*nphp7.2* single knockdown morphants (Movie S16, S17, S18, S19). The movies were recorded from the combined injected morphant embryos showing clear pronephric cysts.
(AVI)
