# Supplementary figures and images for: A Complex of BBS1 and NPHP7 Is Required for Cilia Motility in Zebrafish
Source: PLoS One. 2013 Sep 12;8(9):e72549. doi: 10.1371/journal.pone.0072549 (PMC3771994; doi:10.1371/journal.pone.0072549)

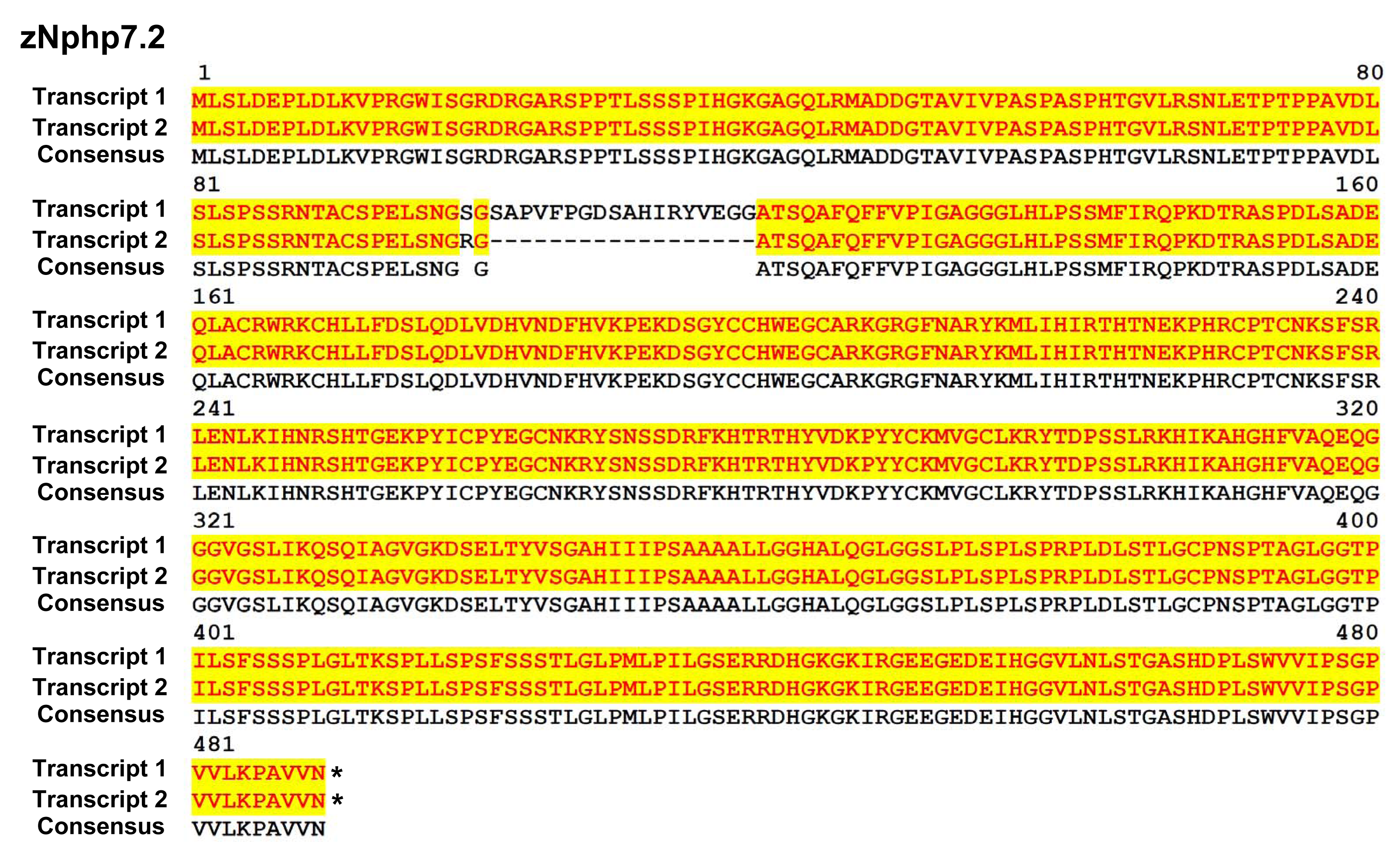

Supplement: Figure S1 — Alignment of the amino acid sequences of the 2 variants of maternally expressed z nphp7.2 transcripts. The alignment shows that 1 amino acid is substituted (S99R) and 18 amino acids are deleted (aa 101–118) in maternally expressed transcript variant 2 (Transcript 2) compared to transcript variant 1 (Transcript 1) of znphp7.2. (TIF) [file pone.0072549.s001.tif]

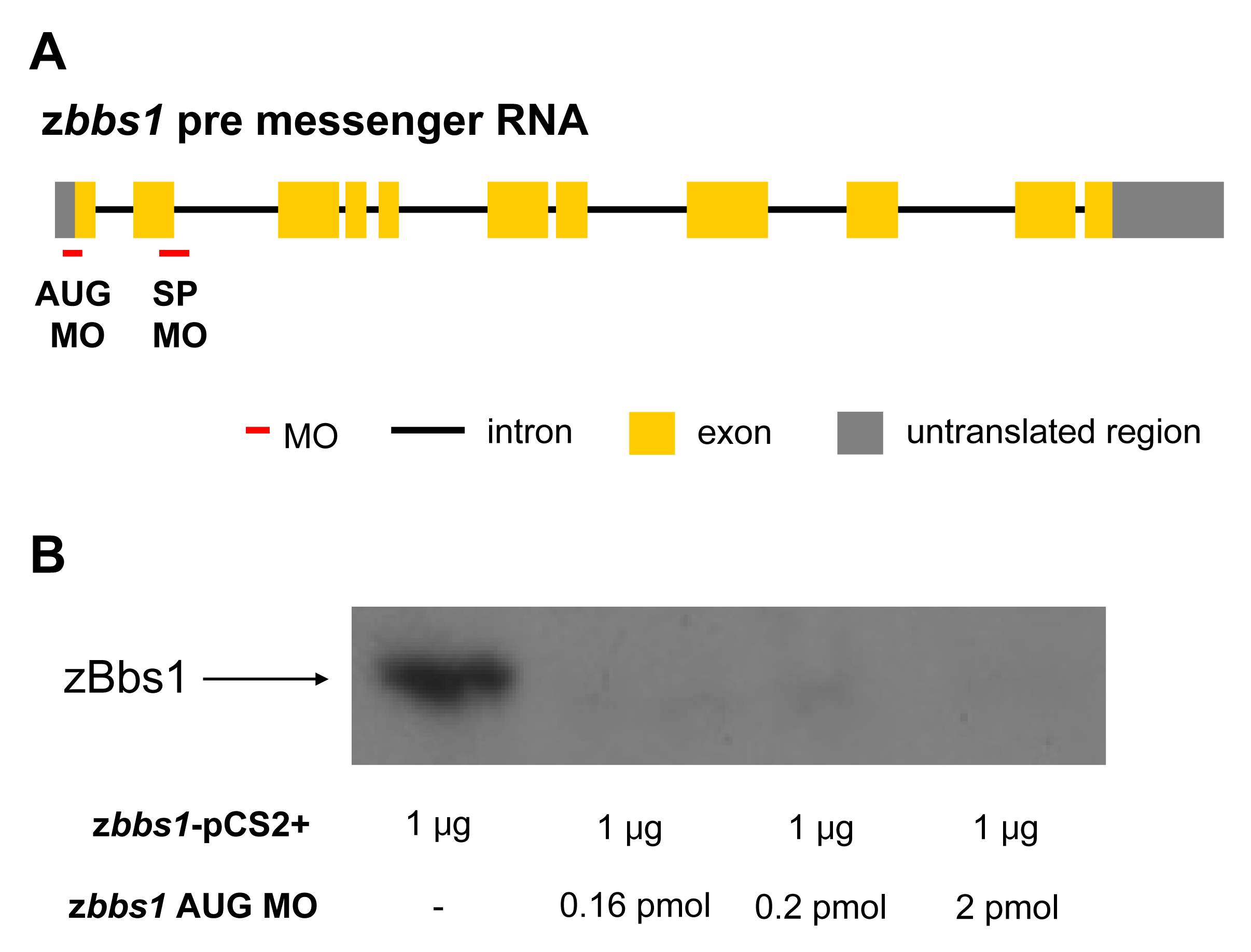

Supplement: Figure S2 — Antisense morpholino oligonucleotides against z bbs1 . (A) MO (red line) targeting zbbs1 translational start codon (AUG MO) and MO targeting exon 2 splice donor site (SP MO) of zbbs1. (B) 1 µg of plasmids DNA containing wild-type full-length zbbs1 in pCS2+ vector was mixed with or without MOs in vitro translation reactions. zbbs1 AUG MO efficiently interfered with zBbs1 protein expression. (TIF) [file pone.0072549.s002.tif]

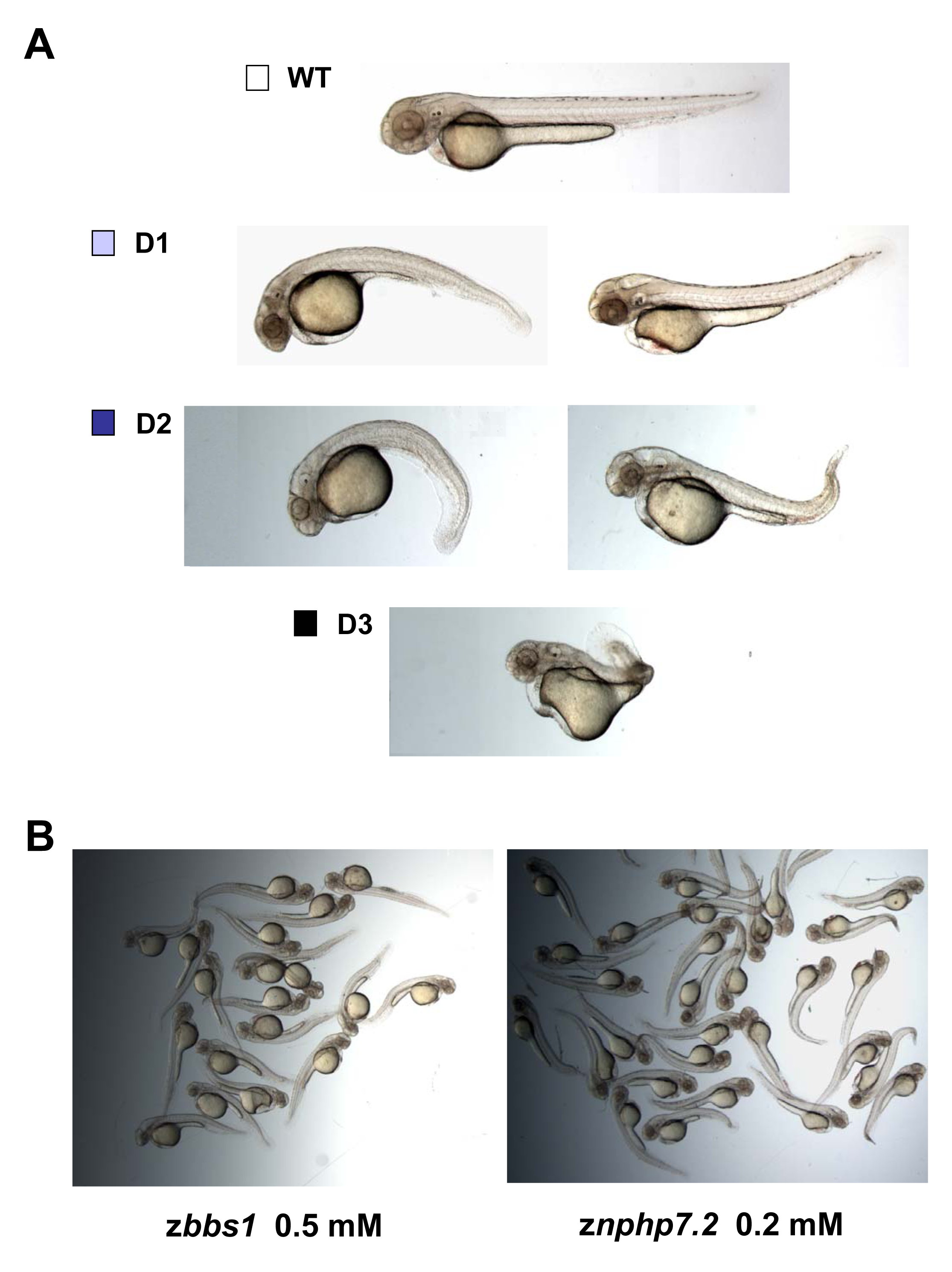

Supplement: Figure S3 — Grading of dysmorphy. (A) Embryos at 48–55 hpf were assessed and scored as wild-type-like or with a degree of dysmorphy ranging from mild (D1) to severe (D3). (B) zbbs1 or znphp7.2 morphant embryos were always consist of a mixed population of individuals with dorsally or ventrally curved body axis. (TIF) [file pone.0072549.s003.tif]

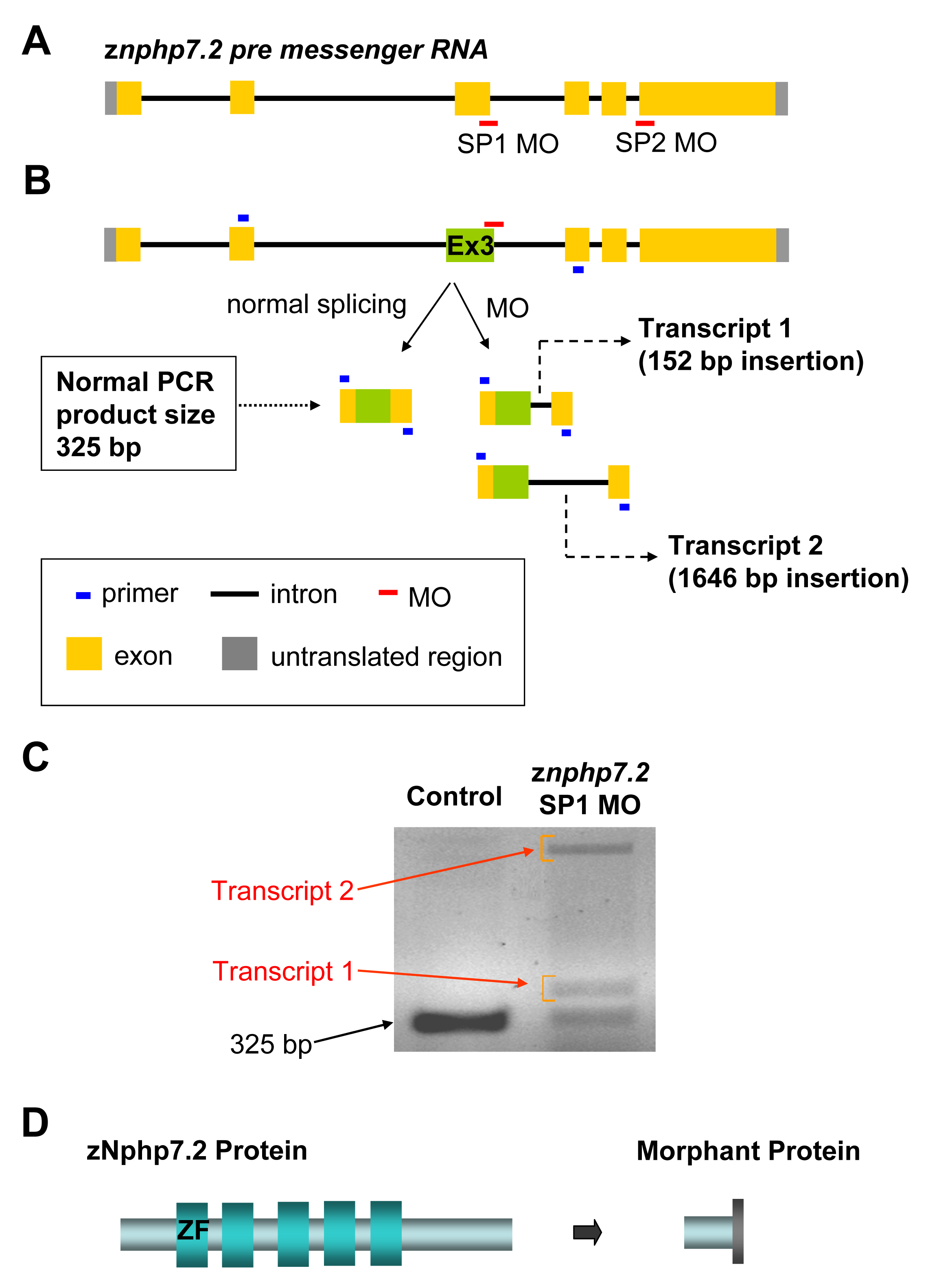

Supplement: Figure S4 — Antisense morpholino oligonucleotides against z nphp7.2 . (A) 2 independent MOs against exon 3 donor site (SP1 MO) and exon 6 acceptor site (SP2 MO) of znphp7.2 were designed. (B and C) RT-PCR was performed with znphp7.2 SP1 MO-injected embryos at 55 hpf. The following PCR with the primers (short blue lines) designed to produce 325 bp amplicon of coding sequence of wild-type znphp7.2 cDNA was performed. znphp7.2 SP1 MO efficiently interfered with normal splicing to cause the insertion of intron sequence between exon 3 and exon 4 resulting in 2 abnormally large amplicons. (Exon 3 is marked as green to visualize the mRNA splicing process.) (D) These abnormal splicing products have a stop codon in the intronic sequence leading to a truncation within the first ZF domain after translating 6 extra amino acids from intronic sequence. This eliminates all other ZF domains together with the C-terminus of zNphp7.2. (TIF) [file pone.0072549.s004.tif]

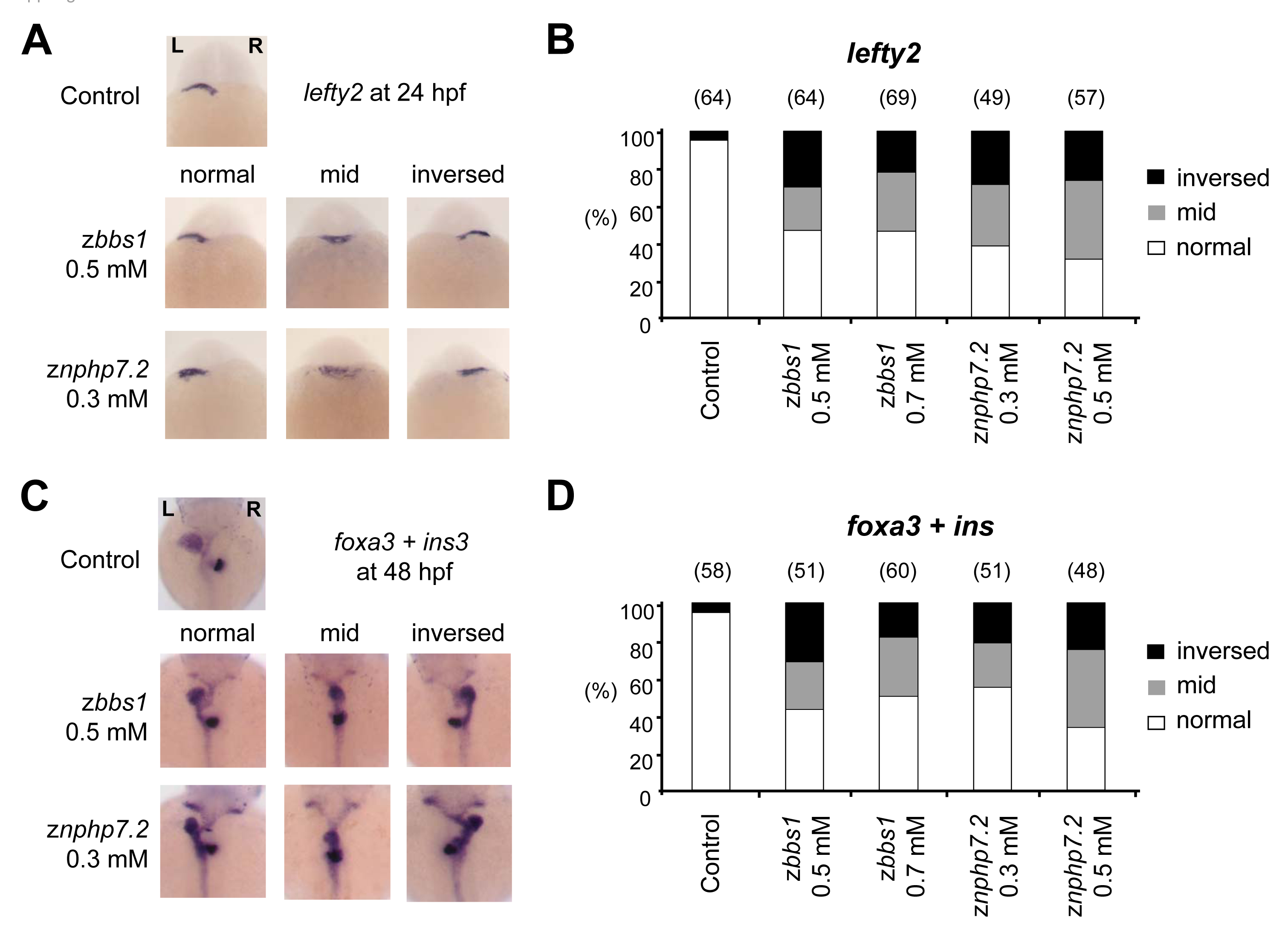

Supplement: Figure S5 — zBbs1- or zNphp7.2-deficient embryos showed defects in organ laterality. (A, B) In situ hybridisation of both zbbs1 and znphp7.2 morphants at 24 hpf with lefty2 probe showed defective left-right asymmetry patterning. (C and D) Defective laterality of liver (foxa3) and pancreas (ins) was observed in zBbs1- and zNphp7.2-depleted embryos at 48 hpf. (TIF) [file pone.0072549.s005.tif]

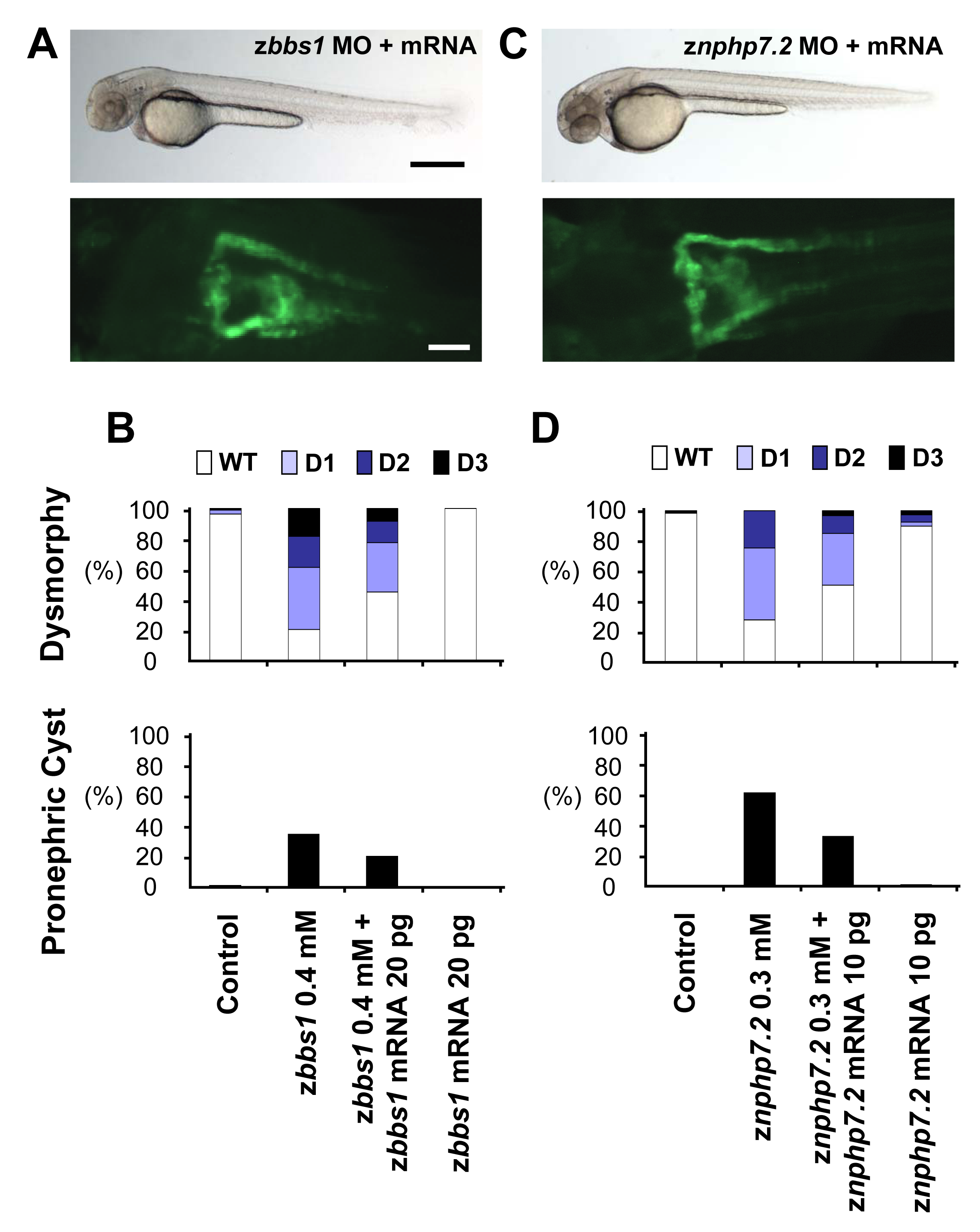

Supplement: Figure S6 — Co-Injection of z bbs1 or z nphp7.2 mRNA partially rescued the corresponding morphant phenotype. Co-injection of 20 pg of zbbs1 mRNA (A and B) or 10 pg of znphp7.2 mRNA (C and D) together with the corresponding MO partially rescued the dysmorphic changes caused by the MO-mediated knockdown, decreasing the dorsal body curvature and rescued pronephric cyst formation. (A: scale bar = 500 µm, B: scale bar = 100 µm) (D, X2 = 5.27, P = 0.022; H, X2 = 14.5, P<0.001). (TIF) [file pone.0072549.s006.tif]

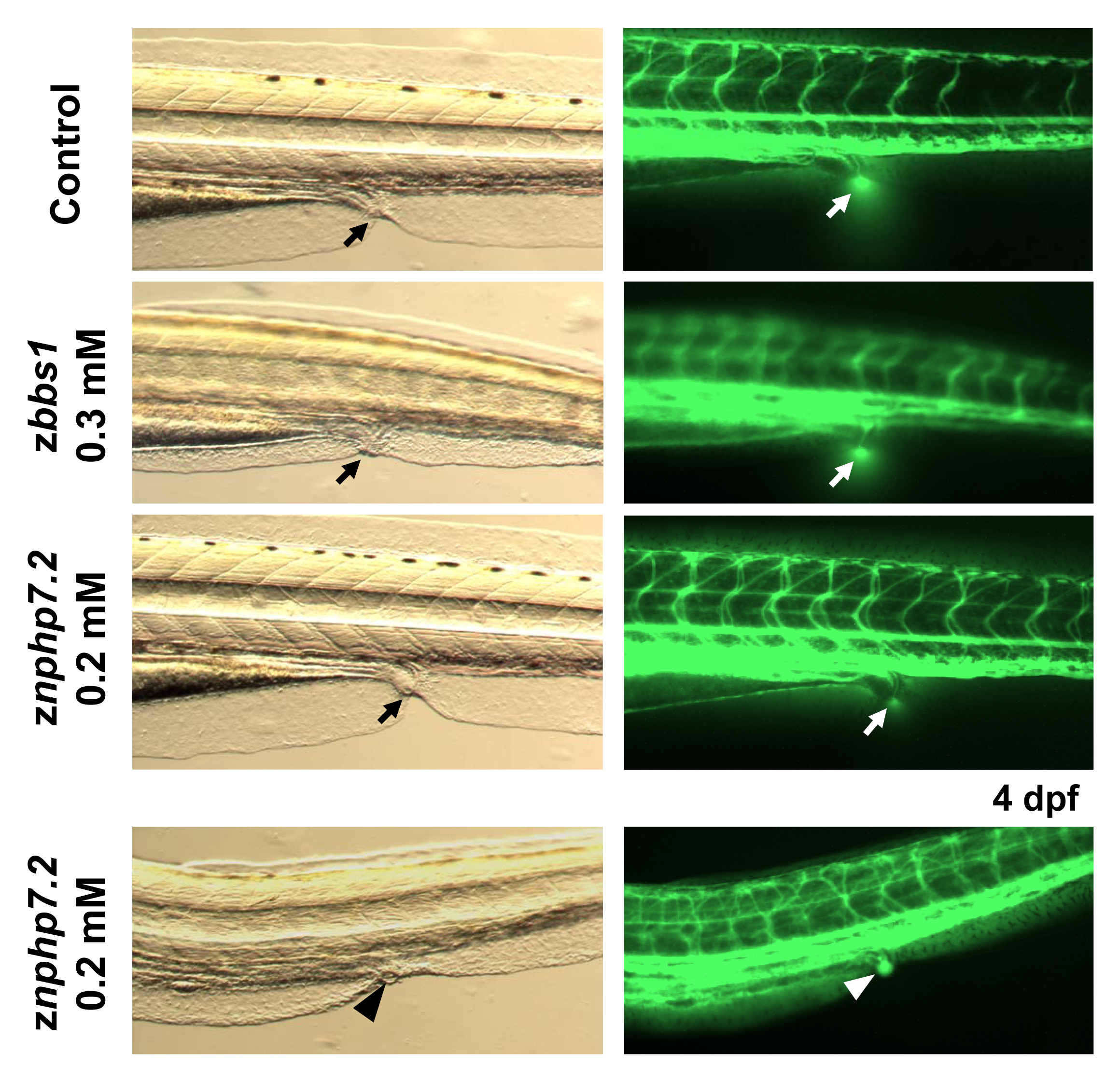

Supplement: Figure S7 — Dextran injection revealed fluid excretion via the cloaca in z bbs1 and z nphp7.2 morphants. Zebrafish control and morphant embryos with pronephric cysts at 96 hpf were injected with 5% FITC-conjugated dextran solution (70 kD) into the circulation. Fluorescent dye excretion with the urine at the cloaca (black and white arrows) was observed in control embryos (22/22), zbbs1 (16/16) and znphp7.2 (21/23) morphants. The lower panel represents a znphp7.2 morphant embryo with missing fluorescent dye excretion due to persistent closure of the cloaca (arrowheads). Images on the left column represent transmitted light images and images on the right column represent fluorescent images of the same embryo for each setting. (TIF) [file pone.0072549.s007.tif]

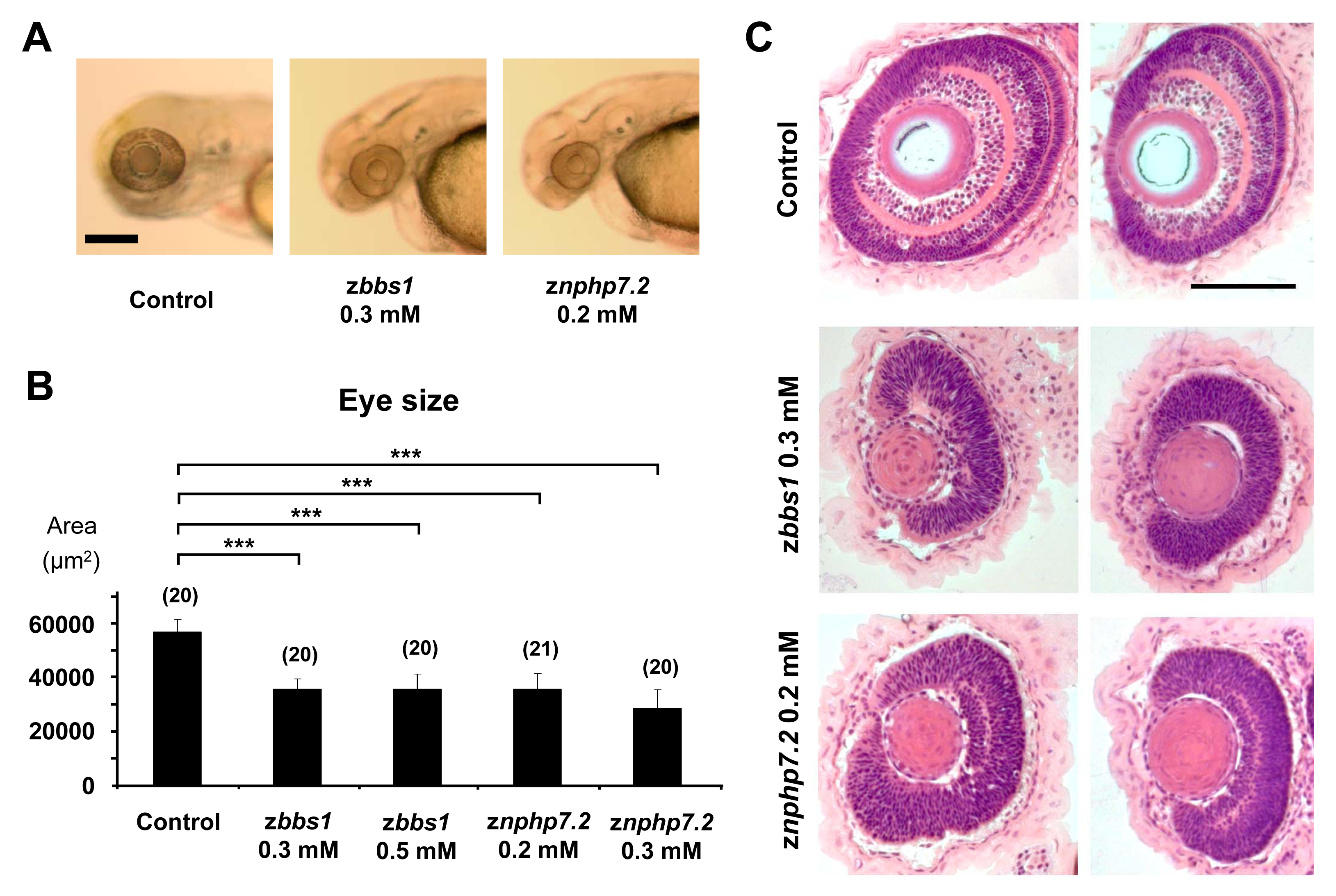

Supplement: Figure S8 — z bbs1 and z nphp7.2 morphants display reduced eye size and defective retinal layer formation. The eye size as area (µm2) was measured for control embryos, zbbs1 and znphp7.2 morphant embryos at 80 hpf. (A) Representative brightfield images showing reduced eye size for zbbs1 and znphp7.2 morphants in comparison to the control (Scale bar = 200 µm). (B) Statistical quantification of the measurements proved that the reduction in eye size for zbbs1 and znphp7.2 morphants was significant in comparison to the control. (C) Histological cross-sections of 96 hpf zbbs1 and znphp7.2 morphants revealed defective layer formation in comparison to the control. Two representative images are shown for each setting. (Scale bar = 100 µm). (TIF) [file pone.0072549.s008.tif]

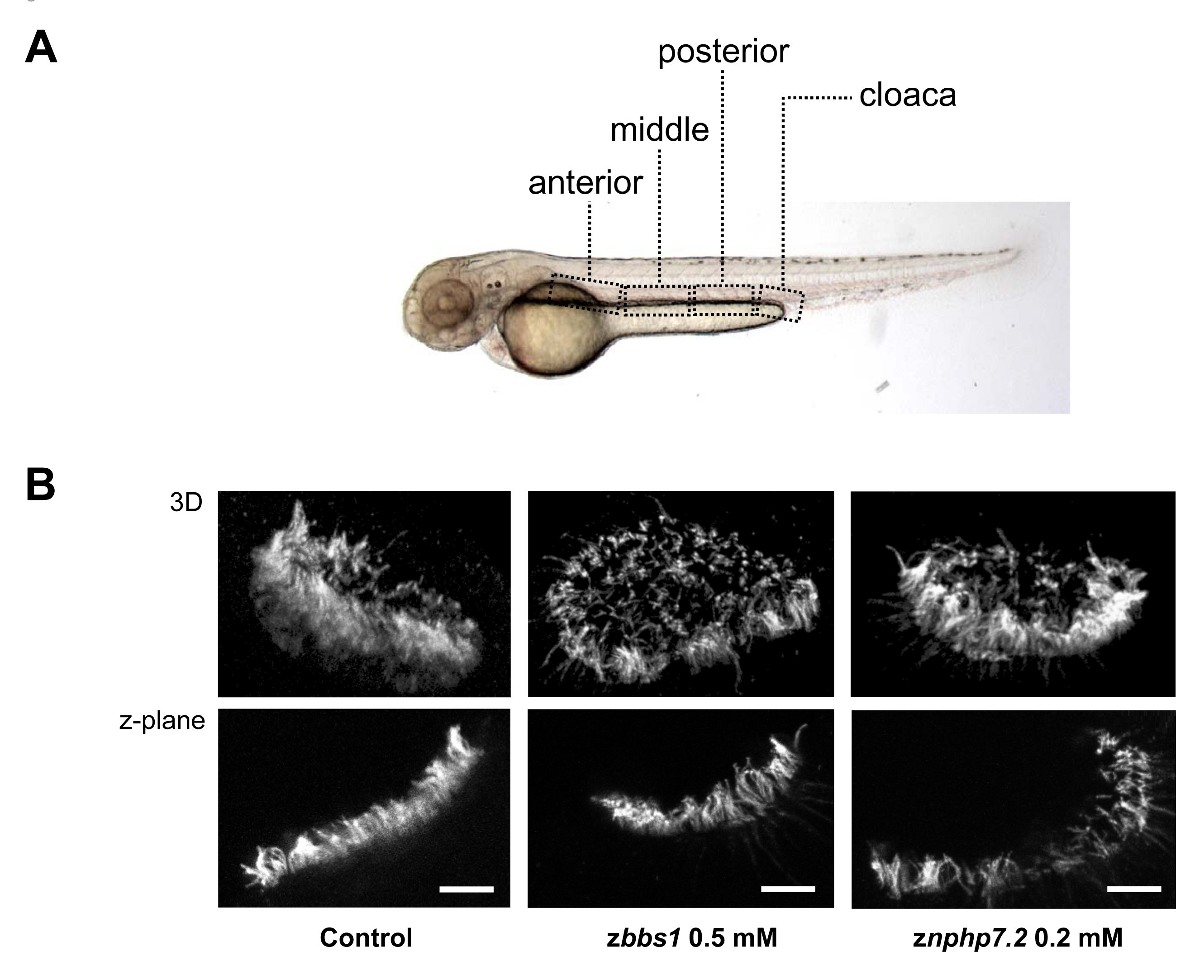

Supplement: Figure S9 — The morphants of z bbs1 and z nphp7.2 exhibited impaired motility of cilia in the pronephric tubule. (A) The areas where the movies were recorded are shown by the dashed box. (B) Acetylated tubulin staining demonstrated normal development of cilia in the nasal pit (Scale bar = 10 µm). 3-dimensional (3D) images (upper panel) and z-plane images (lower panel) show that the cilia formation in the morphants of zbbs1 and znphp7.2 is normal compared to control embryo. (TIF) [file pone.0072549.s009.tif]
